# Dynamic Gene Expression and Alternative Splicing Events Demonstrate Co-Regulation of Testicular Differentiation and Maturation by the Brain and Gonad in Common Carp

**DOI:** 10.3389/fendo.2021.820463

**Published:** 2022-02-08

**Authors:** Yuanli Zhao, Kuangxin Chen, Fei Liu, Mouyan Jiang, Zonggui Chen, Huijie Chen, Yanlong Song, Binbin Tao, Xuefan Cui, Yongming Li, Zuoyan Zhu, Ji Chen, Wei Hu, Daji Luo

**Affiliations:** ^1^State Key Laboratory of Freshwater Ecology and Biotechnology, Institute of Hydrobiology, The Innovative Academy of Seed Design, Hubei Hongshan Laboratory, Chinese Academy of Sciences, Wuhan, China; ^2^University of Chinese Academy of Sciences, Beijing, China; ^3^College of Fisheries, Guangdong Ocean University, Zhanjiang, China

**Keywords:** transcriptome, alternative splicing events, testicular differentiation, testicular maturation, dynamic gene expression

## Abstract

The common carp (*Cyprinus carpio*) accounts for approximately 10% of the annual freshwater aquaculture production and is an ideal model to study cyprinidae reproduction. Female common carp grow faster than the males; therefore, related research presents an opportunity with high application value. Although we have a detailed understanding of common carp’s early gonadal differentiation process, information about genome-wide gene expression, regulation, and underlying molecular mechanisms during this process remain limited. Here, time-course data comprising six key stages during testicular differentiation and maturation were investigated to further understand the molecular mechanisms underlying the testicular development in cyprinid species. After integrating these time-series data sets, common carp genome, including 98,345 novel transcripts and 3,071 novel genes were re-annotated and precisely updated. Gene co-expression network analysis revealed that the ubiquitin-mediated proteolysis pathway was essential for metabolism during testicular differentiation in the endocrine system of *C. carpio*. Functional enrichment analyses indicated that genes mainly related to amino acid metabolism and steroid hormone synthesis were relatively highly expressed at the testicular undifferentiation stages, whereas genes associated with cell cycle and meiosis were expressed from the beginning of testicular differentiation until maturation. The dynamics of alternative splicing events demonstrated that exon skipping accounted for majority of the alternative splicing events in the testis and the brain during gonad development. Notably, several potential male-specific genes (*fanci* and *sox30*) and brain-specific genes (*oxt*, *gad2*, and *tac1*, etc.) were identified. Importantly, we traversed beyond the level of transcription to test for stage- and gonad-specific alternative splicing patterns between the brain and testis. This study is the first to describe a comprehensive landscape of alternative splicing events and gene expression patterns during gonadogenesis in common carp. This work is extremely valuable to elucidate the mechanisms underlying gonadal differentiation in Cyprinidae as well as other fish species.

## Introduction

Carp (cyprinids) contribute over 2 million metric tons to fish production in China, accounting for approximately 10% of the total freshwater aquaculture production (1). They have emerged as the most economically viable teleost family. As a dominant cyprinid species, common carp, *Cyprinus carpio*, has been reported to account for almost 10% of annual freshwater aquaculture production (2). Female carp grow faster than the males; therefore, the mechanism of sex differentiation and gonadal maturation is an intriguing topic that is essential for breeding. Previously, we have reported the detailed process of early gonadal differentiation in morphogen and several other sex-related genes (3). However, information of genome-wide gene expression, gene regulation, and molecular mechanism during gonadal differentiation and maturation remains limited.

Similar to other vertebrates, the major organs involved in fish reproduction are the hypothalamus, the pituitary, and the gonads (testis or ovary). The hypothalamic-pituitary-gonad (HPG) axis regulates the production of sex-related hormones, thereby controlling maturation and spawning. In detail, the brain gonadotropin-releasing hormone (GnRH) stimulates the secretion of pituitary gonadotropins, follicle-stimulating hormone, and luteinizing hormone, which in-turn stimulate the production of sex steroid hormones in the gonads (4). The first report where the HPG axis was employed in aquaculture research involved using the human chorionic gonadotropin (hCG) to initiate spermatogenesis and spermiation in cultivated male Japanese eels (5). Recently, another interesting discovery showed that knocking out *cyp17a1* ensured development into males irrespective of whether they were female or male zebrafish initially without affecting their reproduction ability. A subsequent comparative transcriptomic analysis of control and *cyp17a1* knockout male zebrafish revealed incomplete masculinization and significantly altered expression of brain genes in *cyp17a1* knockout fish (6). These studies focused on several hormones and genes in the reproductive endocrine system. However, almost no studies have investigated the dynamic alternations in genome-wide gene expressions and the underlying biological changes involved in the HPG axis during the gonadal developmental stage, especially the integrated analysis of mRNA expression as well as the processing between brain and gonad.

Alternative splicing mechanisms allow organisms to sense and react to minute changes during early embryogenesis, not only in mammals (7) but also in fish (8). Alternative splicing has also been implicated in various aspects of organogenesis, including testis and ovary development. Previous studies have also identified several stage-biased isoforms from alternative splicing in the developing gonads (9–11), whereas others have identified sex-specific splicing differences in human brains (12). Although various alternative splicing events have been long to known to take place in both the gonad and brain, not much is known about the effects of alternative splicing on gonad development, with even lesser information about the difference in splicing between the gonad and brain. In vertebrates, various synchronous physiological events are required to regulate reproduction, and the molecular mechanism controlled by a comprehensive and interconnected biological system including the brain, the pituitary, and the gonads (13). However, information about the involvement of the HPG axis at the level of alternative splicing is limited, and even less is known about its effects at this level during testicular development. Understanding the alternative splicing landscape of the reproductive axis changes will not only provide more insight into testicular development, but also deepen our existing knowledge about RNA processing and stage-specific alternative splicing events during reproduction.

Transcriptome sequencing (RNA-seq)-based high-throughput sequencing can simultaneously identify genome-wide gene expression and alternative splicing events changes (14, 15). Using the time-course reproductive model and rising genomics models of common carp (16, 17), a strand-specific RNA-seq strategy was employed to analyze the transcriptome of the common carp in developing testis at the six key developmental stages that encompass the period from testicular undifferentiation to maturation. In this study, we delved deeper than the level of transcription to test for stage-specific and gonadal-specific alternative splicing in patterns in the brain and testis of common carp. These time-series data described a comprehensive landscape of the transcriptional events at both the gene and transcript level as well as the underlying mechanisms of sex differentiation and gonad maturation, in addition to providing detailed information that can improve the quality of the existing reference genome annotation.

## Materials and Methods

### Ethics Statement

All experiments were conducted according to the guidelines and regulations outlined by the ‘Management and Use of Laboratory Animals of Hubei Province’ and complied with China’s existing laws and regulations for biological research. This study did not involve any endangered or protected species.

### Sample Collection and Preparation

All experimental common carp were reared at the Guanqiao Experimental Station, Wuhan, China. The XY all-male common carp were produced following the technical procedure shown in **Figure S1** (18). After hatching, the XY all-male carp (about 1000 each) were reared in uniform-sized fishponds at the Guanqiao Experimental Station. Based on the results of our previous report about the time of sex determination and sex differentiation of common carp (3) 26 samples from testis development stages 1 through 6 were prepared for subsequent analyses. Stage 1 to stage 6 were sampled at each of the following days post hatching (dph) sampling points: 25, 35, 70, 100, 120, 180 dph. Given their small size, gonadal tissues collected from stage 1 and stage 2 were stored in TRIzol at -80°C until processing. At other sampling time points, the left side of the collected gonadal tissues were stored in TRIzol at -80°C for total RNA extraction, whereas the right side of the collected gonadal tissues from the same fish were fixed in Bouin’s solution for 12 h, transferred to 70% alcohol, and then used in hematoxylin and eosin (H&E) staining and immunofluorescence experiments.

### Histology and Immunofluorescent Analysis

Gonadal samples extracted from stage 1 to stage 6 were fixed in 10% neutral buffer formalin at 4°C, dehydrated in a graded ethanol series, leaned in xylene, embedded in paraffin, and then cut into 4 μm sections using a rotary microtome (Leica, Germany). The sections were stained with H&E for routine histological examination and observed using the light microscopy (Olympus BX51, Japan).

Double immunofluorescent assays were performed using 4-μm paraffin sections stained with anti-mouse VASA (1:500 dilution; 2008, DIA-AN, Wuhan, China) and anti-rabbit SCP3 (1:500 dilution; Ab150292, Abcam, UK) as the primary antibodies, as well as Dylight488 anti-rabbit IgG (1:1000 dilution, Abbkine, CA, USA) and Dylight594 anti-mouse IgG (1:1000 dilution, Abbkine, CA, USA) as the secondary antibodies. VASA and SCP3 are reliable markers for germinal cells and meiotic germ cells in several animals, including zebrafish (19), Nile tilapia (20), Gibel carp (21). Therefore, germ cells and spermatocytes can be easily detected during early development by examining the immunofluorescence for fish VASA and SCP3 proteins. Prior to blocking, the same histology procedure as that described previously was employed; following which the sections were blocked using 2% BSA (Sigma, USA). Subsequently, sections were incubated overnight with the primary antibodies at 4°C. The next day, these sections were incubated along with in the secondary antibodies for 1 h, and then washed with PBS and 4’, 6-diamidino-2-phenylindole (DAPI) (Sigma, USA) for nuclear staining. Sections processed without the anti-mouse VASA were used as a negative control. Finally, images were acquired using a Leica TCS SP8 confocal system (Leica Microsystems, Wetzlar, Germany) and analyzed using the LAS AF Software.

### RNA Isolation, cDNA Library Preparation and Sequencing

The total RNA was extracted from 11 brain tissues samples (2 from the stage 3, 3 from the stage 4, 3 from the stage 5, and 3 from the stage 6) and 15 testis tissues samples (3 from the stage 1, 2 from the stage 2, 2 from the stage 3, 2 from the stage 4, 3 from the stage 5 and 3 from the stage 6) using TRIzol Reagent (Invitrogen, USA), and then treated with RNase-free DNase I (Thermo Scientific, USA) at 30°C for 30 min to remove genomic DNA contaminants. Subsequently, the purity, quantity, and integrity of the extracted total RNA samples were measured using a NanoDropND-2000 spectrophotometer (Thermo, Waltham, USA), 1.2% (w/v) agarose gel electrophoresis, and an Agilent 2100 Bioanalyzer (Agilent Technologies, Richardson, USA), respectively. RNAs with an RNA Integrity Number (RIN) > 8, 28S/18S > 0.7, and A260/280 values of approximately 2.0 were used to construct the RNA-seq library. Poly (A) mRNAs were isolated from the total RNAs using poly (dT) oligo-attached magnetic beads, and cDNA libraries were prepared using the TruSeq RNA Sample Preparation Kit (Illumina, USA). In total, the 26 cDNA libraries were sequenced using the Illumina Nova-seq (BGI) sequencing platform to generate 150bp pair-end reads.

### Transcriptome Assembly and Annotation

The raw data generated from Illumina sequencing were filtered based on quality to remove adaptor sequences, short reads with length of less than 10 bp, and low-quality reads (i.e., the percentage of bases with quality values less than 5 exceed 50% in the read) using fastp (22). The filtered reads were first aligned with the common carp genome using STAR (v2.7.2b) with two-pass mode mapping. Then, these reads were assembly for a single sample using StringTie (23) and all samples were merged using TACO (24). All genes were annotated in the genome sequences available in the six public databases, including NCBI non-redundant protein sequences (Nr), Swiss-Prot, Kyoto of Encyclopedia of Genes and Genomes (KEGG) (25), Gene Ontology (GO) (26), Non-supervised Orthologous Groups (eggNOG) (27), and Pfam (28).

### Differential Alternative Splicing Analysis and Validation

Sequencing reads were mapped to the assembly transcript using the STAR aligner v2.7.2b in the two-pass mapping mode. The differential alternative splicing (AS) events were identified based on assembled transcript sequences obtained from the RNA-Seq sequencing using rMATS with default parameters (29). In order to validate the accuracy of the AS detected with RNA-Seq, PCR was conducted for six selected genes, *robo2*, *epb41l3b*, *cadm3*, *cadm4*, *macf1*, and *ptprfa*. The total RNA from the testes and brain samples was extracted as described above. The PrimeScript II 1^st^ Strand cDNA Synthesis Kit (TaKaRa, Japan) and SYBR Premix Ex Taq II (TaKaRa, Japan) were used for reverse transcription reaction and the PCR assay. Specific primers (**Supplemental Files 1**: **Table S1**) for the selected genes were designed using NCBI Primer-BLAST (NCBI, USA) according to the homologous flanking sequences or specific splicing of exons for all potential alternative splicing transcript isoforms. The PCR amplification cycle was as follows: 35 cycles at 95°C for 15 s, 57°C for 15 s, and 72°C for 15 s followed by 72°C for 3 mins. PCR products were examined and isolated on a 1% agarose gel.

### Co-Expression Gene Network Analysis

The weighted gene co-expression network analysis (WGCNA) R package, version 3.6.3 (30, 31) was used to construct co-expression networks for the entire dataset, containing 45,393 genes. A correlation matrix was constructed for all relevant samples using the bi-weight mid-correlation between all genes. The soft-thresholding power, β, was maintained at 6 and used to derive an adjacency matrix that exhibited approximate scale-free topology (R^2^ > 0.85). The adjacency matrix was transformed into a topological overlap matrix (TOM). The matrix 1-TOM was used as the input to calculate co-expression modules using hierarchical clustering. Modules were branches of the hierarchical cluster tree base, with the minimum module size set to 20 genes. Modules with similar expression profiles were merged by hierarchical clustering of the gene correlation eigen values (correlation > 0.80). Genes with no network correlation were placed into the module Grey.

The genes contained in the most significant module were submitted for analysis of functional enrichment using the clusterProfiler (version 3.9), which included Gene Ontology (GO) and Kyoto Encyclopedia of Genes and Genomes (KEGG) pathway terms (32). *P*-value lower than 0.05 indicated statistical significance, and the top 10 terms were selected for visualization. For the most significant module (‘lightcyan’), the gene-gene interaction network was constructed and visualized using Cytoscape v3.8.2. WGCNA provided the weightage for the interacting genes. In a given network, each gene is represented as a node and the interactions between the nodes are defined as edges. The degree is defined by the number of edges connected to a node, and nodes with a high degree correspond to the hub genes that likely have important biological functions. Nodes with carrying more interactions were considered the hub genes.

### Different Gene Expression Analysis and Enrichment Analysis

Differentially expressed genes (DEGs) between the brain and the testes at each of the six developmental stages as well as those between consecutive stages were identified based on Baggerly’s test on Fragments Per Kilobase of exon per Million mapped fragments (FPKMs). Benjamini-Hochberg correction (33) was used to adjust the original P-values in Baggerly’s test to minimize the false discovery rate (FDR). A DEG is declared if the associated P_FDR_ < 1e-7 and an absolute value of log2(fold change) > 1 were regarded as the cut-off criteria. The clustering analysis was conducted using Pheatmap package in R based on FPKM of DEGs. Then DEGs were enriched and analyzed with GO term (34) and KEGG pathway using clusterProfiler (35).

### Validation by Quantitative Real-Time PCR (qRT-PCR) Analysis

In total, 15 genes associated with testes development were randomly selected for qRT-PCR to validate the RNA-seq results. Reversed transcribed cDNA obtained from the total RNA used for transcriptome sequencing was synthesized using the PrimeScript™ RT reagent Kit with gDNA Eraser (Takara, Shanghai, China) according to the manufacturer’s protocol. All cDNA samples were diluted to 5 ng/µl and stored at −80°C until use. Specific primers, listed in **Table S2**, were designed based on the NCBI Primer-BLAST (NCBI, USA). All qRT-PCR reactions were performed in triplicates and target specificity was determined based on the dissociation curve analysis. β-actin was selected as the internal control to normalize each gene’s expression level. The relative expression level of the target gene versus β-actin was calculated using the 2 ^-ΔΔCT^ method. The obtained data were statistically analyzed using GraphPad Prism.

## Results

### Histological and Immunofluorescence Analysis During the Major Stages of Sex Determination, Testis Differentiation and Maturation

**Figure 1:**
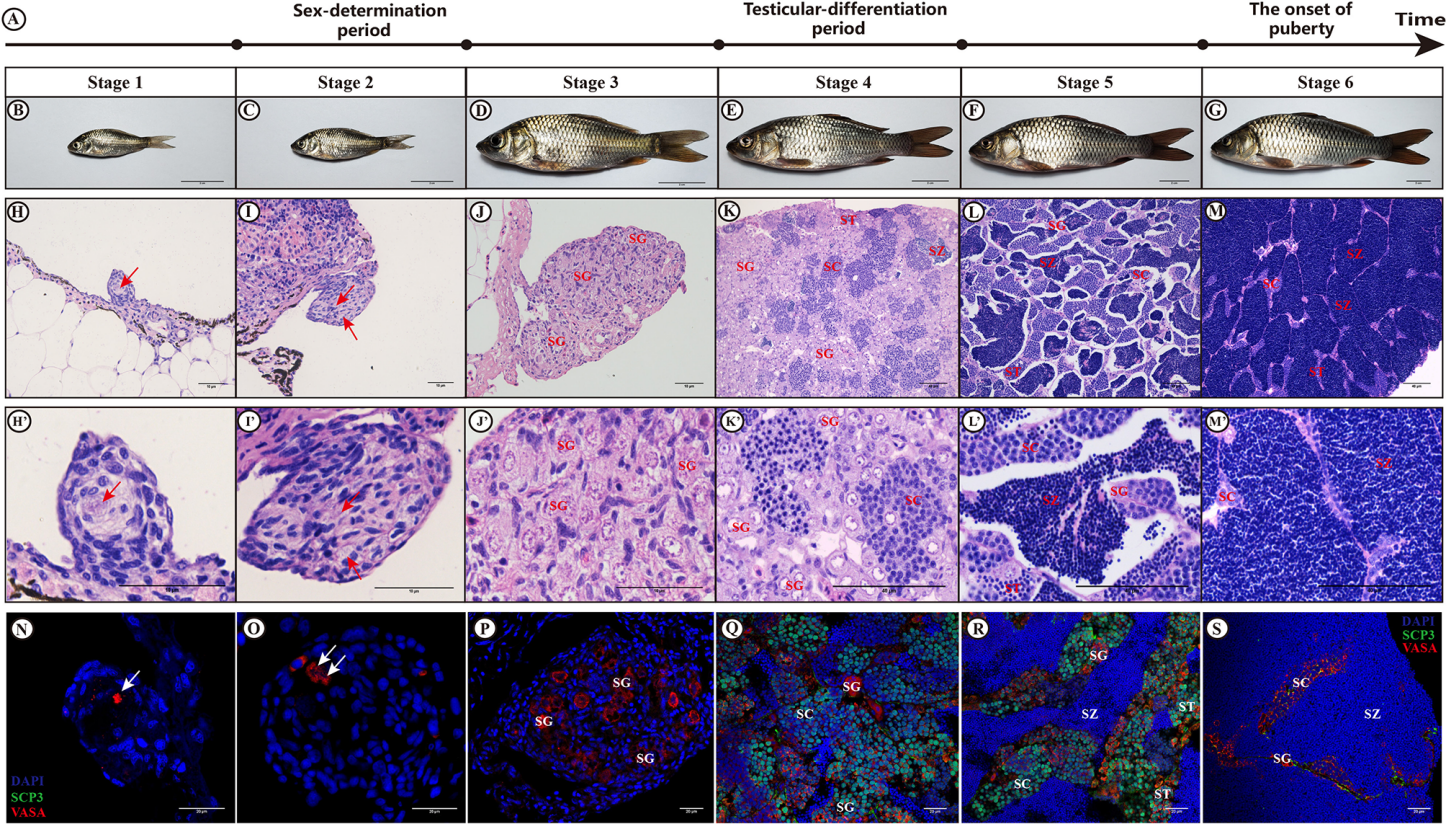
Histology and immunofluorescent analysis. Observation of the gonads of XY male common carp at different developmental stages by H&E staining and immunofluorescence. **(A)** show the diagram illustrating the important biological events that occurred at six different testis developmental stages; **(B–G)** represent the gross morphology of fish samples at stages 1-6; **(H–M)** represent H&E staining results for the gonad at stages 1-6; **(N–S)** refer to the immunofluorescence results after staining with anti-VASA and anti-SCP3 antibodies at stages 1-6; Arrows, PGC; SG, spermatogonia; SC, spermatocytes; ST, spermatids; SZ, spermatozoa; Bar in **(B–G)**, **(H–J)** and **(H'–J')**, **(K–M)** and **(K'–M')**, and **(N–S)** represent 2 cm, 10 µm, 40 µm, and 20 µm, respectively. The sections incubated without primary antibodies did not show any immunostaining (not shown).

Histological (HE) and immunofluorescent (IHC) analysis confirmed the premature and mature stages of the XY male common carp at different time points (**Figure 1**). Based on the HE staining and IHC results at different developmental time points of male gonad reported by Jiang et al. (3), the male gonads were divided into six consecutive developmental stages, named stage 1 to stage 6. Stage 1 and Stage 2 refer to the period before and after sex determination, respectively. Stage 3 represents the active proliferation of spermatogonia, which occurs immediately before the beginning of meiosis and differentiation of testes. Stage 4 represents the formation of the spermatocytes or the prometaphase of meiosis, which were confirmed by double immunostaining VASA and SCP3. Stage 5 indicates the appearance of spermatozoa or the end of the meiotic period. Finally, stage 6 is indicative of full of spermatozoa and represents the finish of spermatogenesis and also the onset of puberty.

### *De Novo* Assembly and Characterization of the *C. carpio* Transcriptomes

**Figure 2:**
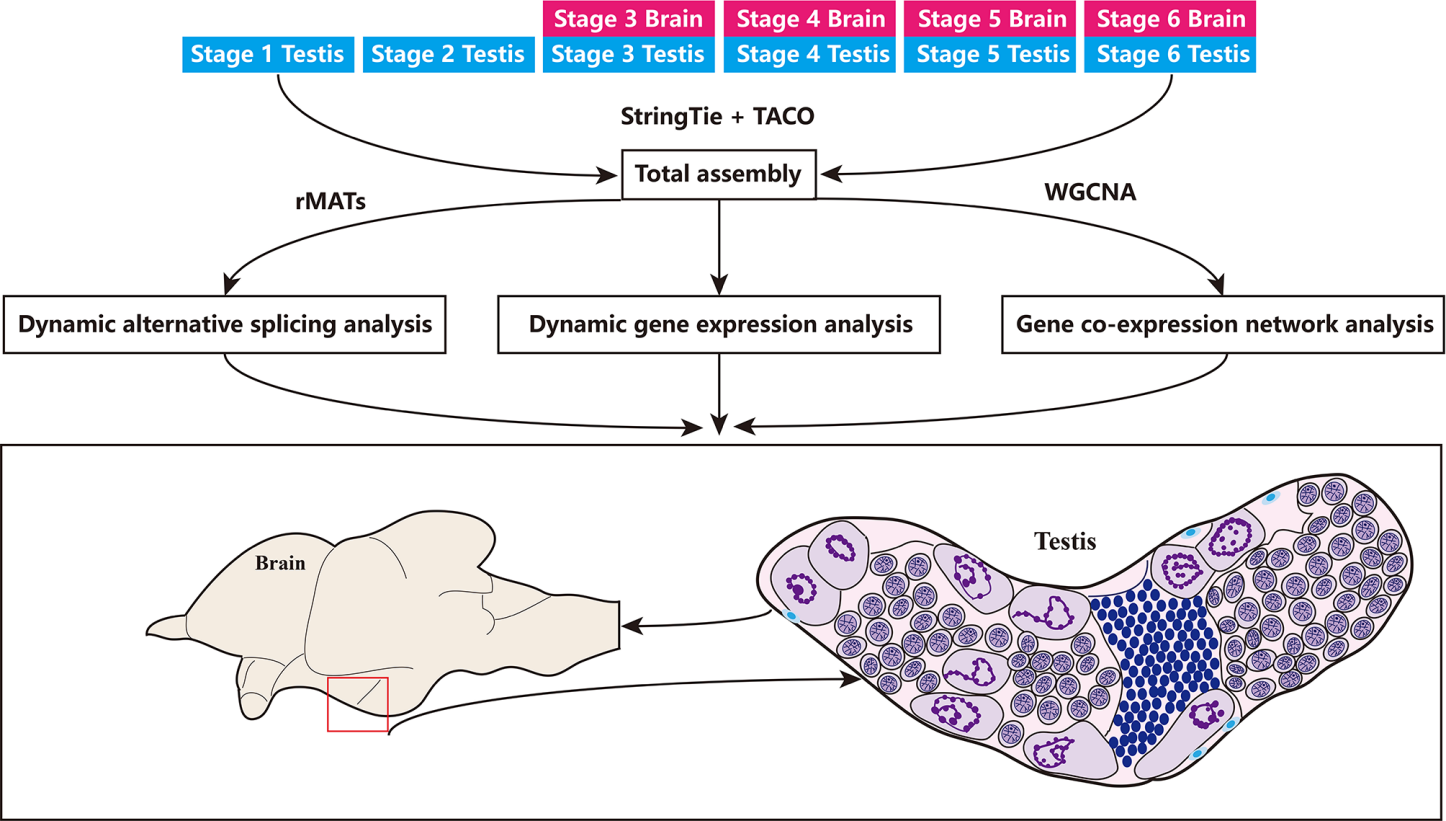
Overview of the study. Samples used are listed on the top, including 4 stages in the brain and 6 stages in the testes. This data set was first used for transcriptome assembly, then for dynamic gene expression quantification and alternative splicing analysis, as well as for gene co-expression network analysis in the brain and testes.

**Figure 3:**
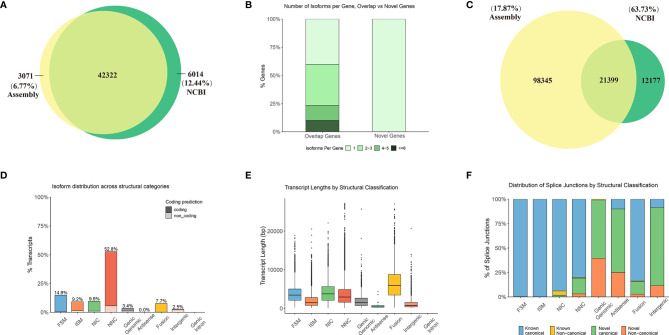
Further detailed annotation of the common carp genome through the transcriptome of the testes and the brain at different developmental stages. Venn diagram depicting 42,322 overlapping genes between the NCBI data and the assembly. **(A)** Venn diagram of the number of gene annotations comparisons between assembly and NCBI data. **(B)** Number of isoforms per gene in overlapping and novel genes. **(C)** Venn diagram of the number of transcript annotations between the assembly and NCBI data. **(D–F)** Characteristics of the annotated transcripts. FSM, full splice match; ISM, incomplete splice match; NIC, Novel in catalog; NNC, Novel not in catalog. **(D)** Isoform distribution across structural categories. **(E)** Transcript lengths by structural classification. **(F)** Distribution of splice junctions based on structural classification.

Gonadal samples were collected at six key stages from stage 1 to stage 6, whereas brain tissue was collected at four key stages from stage 3 to stage 6 (**Figure 2**). For all surveyed time points, two or three replicates were collected. In total, 26 samples yielded 120 million raw reads by RNA-seq, which were deposited into the NCBI SRA repository under the accession number (PRJNA781298). After filtering for quality, 109.1 million clean reads were obtained and assembled into 119,744 transcripts and 45,393 unigenes, including 42,322 unigenes that overlapped with genes in the NCBI database, and 3,071 novel unigenes (6.77%) (**Figure 3A**). Although numerous isoforms existed for each overlapping gene, ranging from 1 to 6; however, the novel genes all existed as a single isoform (**Figure 3B**). For genes present in 2 to 5 isoforms, our assembly results were slightly better than those reported in the NCBI database (**Supplementary Figure S2A**). Of the 119,744 transcripts in our assembly, only 21,399 transcripts (17.87%) matched with those in the NCBI database (**Figure 3C**). Therefore, this assembly discovered numerous novel isoforms (98,345), providing a wealth of information on alternative splicing in the common carp genome. Most of the assembly corresponded to novel transcripts with multiple exons and at least one junction match with transcripts in the NCBI database (**Supplementary Figure S2B**). Heatmap revealed tissue- or stage-specific expression of transcripts in the brain and the testes at different developmental time points (**Supplementary Figure S2C**). 52.8% of the assembly transcripts belonged to the novel not in catalog (NNC), which are new transcripts containing unknown splicing elements (**Figure 3D**). Transcript length analysis and distribution presented in **Figure 3E**, indicated that most transcripts were around 3000 bp in length. Structural classification of NNC revealed that most were known canonical and only a small proportion were the known non-canonical, novel canonical, and novel non-canonical (**Figure 3F**).

### Global Outlook of Alternative Splicing Events Across Gonadal Development

**Figure 4:**
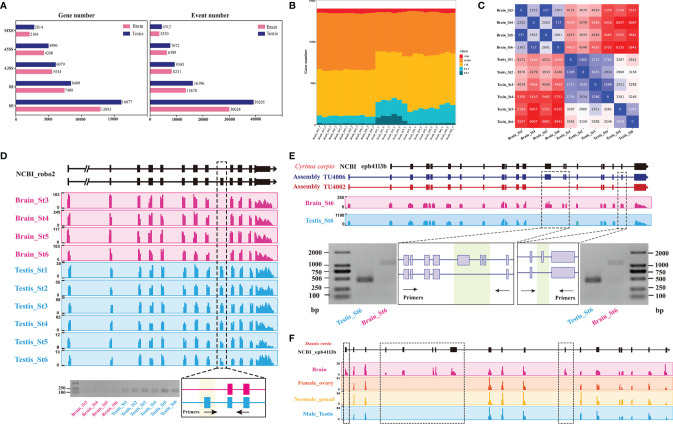
Comparison of the alternative splicing (AS) patterns in the testes and the brain during gonad development of *C. carpio*. **(A)** Statistics of categories of AS events and associated genes identified in the gonad and the brain across time course. A3SS, alternative 3ʹ splice site; A5SS, alternative 5ʹ splice site; RI, intron retention; SE, exon skipping. **(B)** The FPKM expression levels of genes from SE events in the testes and brain at different stages based on RNA-seq. **(C)** Heatmap showing numbers of differential AS events between consecutive time points in either the brain or the gonad. **(D)** The case of the SE pattern of the *robo2* gene and validation using RT-PCR in the testes and brain of *C. carpio*. The expressions of the *robo2* exons are shown with sequence coverage depth on the gene loci. **(E)** The case of the SE pattern of *epb41l3b* gene and its validation using RT-PCR in testes and brain of *C. carpio*. The expressions of the *epb41l3b* exons are shown with sequence coverage depth on the gene loci. **(F)** The case of the SE pattern of the *epb41l3b* gene in the gonad and brain of *Danio rerio*.

An important advantage of RNA-Seq technology is that it allows transcript isoforms to be distinguished and quantitated in an unbiased, genome-wide manner. In order to investigate the changes in alternative splicing (AS) events during time-course gonad development of *C. carpio*, the mRNA splicing patterns at different stages in the testes were compared with those in the brain. Based on the assembled transcript, a systematic analysis of AS was performed for 45,393 genes of *C. carpio*. In total, 76,928 AS events in 84.1% genes from the testis tissues and 61,844 AS events in 71.0% genes from the brain tissue were identified (**Figure 4A**). Our results revealed that skipping exon (SE) accounted for majority of all AS events in the testes (51.0%) and the brain (48.5%) of *C. carpio*. The percentage of the other four AS event patterns in the testes were 21.1, 12.1, 10.0, and 5.9% for intron retention (RI), alternative 3ʹ splice site (A3SS), alternative 5ʹ splice site (A5SS), and mutually exclusive exons (MXE), respectively. In the brain tissues, these AS pattern percentages were 22.1, 13.3, 10.3, and 5.7% for RI, A3SS, A5SS, and MXE, respectively (**Figure 4A**). The FPKM expression level of genes related to all SE events in the testes and the brain were found to be mainly distributed between 1 and 100 (**Figure 4B**). We further characterized the dynamic changes of these SE events in the testes and the brain across gonad development (**Figure 4C**), indicating that the most significant differential SE events occur in the brain rather than in the testes at different stages of gonadal development. For example, a significant SE event occurred in *robo2* in the brain compared with that in the testes during gonad development, which validated the accuracy of the RT-PCR results (**Figure 4D**). Interestingly, we also found multi-exon skipping in the *epb41l3b* gene at the sixth stage in the testes compared to that in the brain of *C. carpio*, which was verified by RT-PCR (**Figure 4E**). Furthermore, multi-exon skipping was also observed in the brain compared with the gonad (ovary, testes, and neo-male gonad) in *Danio rerio* (**Figure 4F**). SE events in *cadm3*, *cadm4*, *macf1* and *ptprfa* in the brain compared with the testes across gonadal development was experimentally verified, as shown in **Supplementary Figures S3**–**S6**. There were also SE events in these five genes (*cast*, *hipk1a*, *dmxl2*, *clasp2*, and *nrf2b*) between the brain and the testes, but they have not been successfully verified by RT-PCR (**Supplementary Figures S7**–**S11**).

### Dynamic Gene Expression During Gonad Development

**Figure 5:**
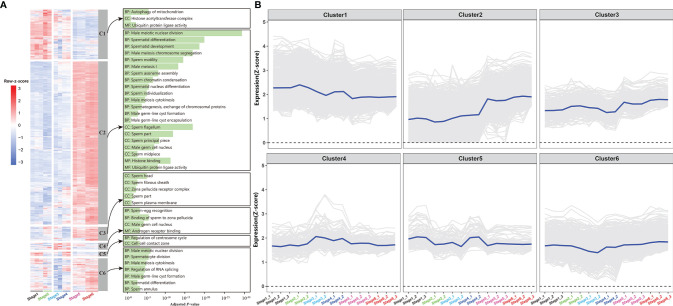
Clustering of expression profiles in the gonad. **(A)** Heatmap of six expression clusters across gonad development. The bar plots on the right display selected GO enrichment of relevant clusters. BP, biological process; CC, cell component; MF, molecular function. **(B)** Time-series expression profile of six clusters. The blue represents the average expression of the cluster, and the background lines represent all genes assigned to this cluster. The expression values were represented by FPKM/z-score.

RNA-sequencing technology was used to obtain gene expression profiles for the testes and brain at the gonadal development stages. In total, expression profiles of all genes during testes development were obtained, two-thirds of which reached at least 1 FPKM (**Supplementary Figure S12**). The cumulative fraction curves revealed that the gene expression distribution was relatively consistent among the 26 samples (**Supplementary Figure S13**). In order to describe dynamic gene expression in the testes, these gene expression profiles were grouped into 6 clusters, some of these clusters were enriched in genes involved in the same biological process (**Figures 5A, B**). GO enrichment analysis of Cluster 2 revealed enrichment in many functions corresponding to the relevant stage (**Figure 5A**). For example, male meiotic nuclear division, spermatid differentiation, and development were found to be highly enriched in Cluster 2, which gradually increased during testes development. In detail, several meiosis-associated genes (*dmc1*, *sycp3*, *spo11*, *mlh1*), male-biased genes (*dmrt1*, *fanci*, *sox30*), and a germ cell maker gene (*piwil*) were identified in the Cluster 2 (**Figure 5B**). Notably, *fanci* is a vital component of the Fanconi anemia pathway, which is crucially involved plays in spermatogenesis of mice and regulates meiotic histone methylation (36), indicating that this gene likely plays an important role in *C. carpio* spermatogenesis as well. Recently, *sox30* is specifically expressed in the testes and plays essential roles in Nile tilapia spermatogenesis. Considering the similarity in *sox30* sequence, similar function could occur in the process of *C. carpio* spermatogenesis (37). Another male-specific gene (*amh*) was identified in Cluster 4 that reached its peak expression level at stage 3. In addition to sex-related genes, several testis steroidogenesis-related genes were identified in the testis, including *hsd17b3* in the Cluster 3 and *hsd11b2* and *cyp11b* in the Cluster 5. The gene expression profile of *hsd17b3* was found to be at its lowest at stage 4, which significantly increased at stage 5 and peaked at stage 6 (**Figure 5B**). The dynamically regulated progression of the brain’ involvement in *C. carpio* testis development was investigated by clustering global gene expression profiles in the brain into 5 clusters (**Supplementary Figure S4**). Notably, genes related to the HPG axis were found in Cluster 1 (*kiss2*, *scg2a*), Cluster 2 (*kiss1r*, *ghrh-lr*, *oxt*, *scg2b*, and *tac1/3*), and Cluster 4 (*gnrh3, gad1*, and *gad2*) (**Supplementary Figure S14**). The clustering information could be useful in identifying genes involved in major biological events and in understanding the manner in which these genes orchestrate in complex gonad development.

### Identifying Modules of Co-Expression in the Testis and Brain

**Figure 6:**
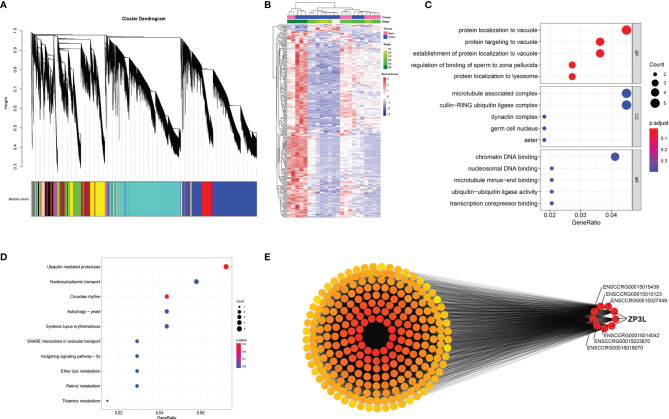
Weighted gene co-expression network analysis (WGCNA) reveals discrete gene networks across testicular development. **(A)** WGCNA cluster dendrogram of time series samples identified genes that were assigned into co-expression modules. Based on similarities in module gene expression, hierarchical cluster tree with showing 33 modules of co-expressed genes. Each of the 48336 genes is represented by a leaf in the tree, and each of the 33 modules by a major tree branch. The lower panel shows modules in designated colors, such as ‘Blue’, ‘Pink’, ‘Turquoise’, and others. Genes within the gray-shaded boxes were not assigned to a module. **(B)** Cluster analysis of genes from the most significant module. Heatmap showing FPKM expression level of these genes. **(C)** The GO enrichment analysis of genes from the most significant module. **(D)** The KEGG enrichment analysis of genes from the most significant module. **(E)** Cytoscape representation of co-expressed genes from the ‘lightcyan’ module.

The correlation coefficients of Testis_St1_3, Brain_St5_3, and Brain_St6_1 were unable to satisfy the requirements of biological repeats within the corresponding intragroup; therefore, these three samples were eliminated and the remaining 23 samples for the subsequent weighted gene co-expression network analysis (WGCNA) (**Supplementary Figure S15**). The best soft-thresholding was determined at a degree of independence of 0.8 (**Supplementary Figure S16**). This WGCNA analysis was performed on 45,393 genes, resulting in identification of 33 modules of co-expressed transcripts (**Figure 6A**). Analysis of the module-trait relationships revealed that the module ‘lightcyan’ was the most highly correlated with stage in the 23 samples (**Supplementary Figure S17**, **S18**). Therefore, the 249 genes from module ‘lightcyan’ were considered to be important in the developmental stage of the testes and brain. Heatmap revealed the FPKM expression level of these 249 genes from the ‘lightcyan’ module (**Figure 6B**).

In order to understand the function and pathway of the most significant stage-correlated co-expression module, lightcyan, was analyzed using clusterProfiler. The top 5 GO terms for biological processes (BP), cell component (CC), and molecular function (MF) are displayed in the dot plots shown in **Figure 6C**, and the top 10 KEGG pathway terms are shown in **Figure 6D**. The GO enrichment analysis of this module was dominated by functional categories related to the control of the male reproduction system, including regulation of the binding of sperm to zona pellucida, and the germ cell nucleus. The KEGG enrichment analysis of this module revealed several pathways involved in protein-related functions, including ubiquitin-mediated proteolysis, nucleocytoplasmic transport, and circadian rhythm. These results indicated that genes in the lightcyan module significantly impact the processes of spermatogenesis and spermatid development, sperm motility, as well as fertilization. Cytoscape representation of the 249 genes from the ‘lightcyan’ module indicated that 9 hub genes were highly connected. Interestingly, 3 of these 9 genes were found to encode zona pellucida sperm-binding protein 3-like (*zp3l*), indicating that this gene is a crucial element influenced by the development of the testes and brain (**Figure 6E**).

### Identification and Enrichment of Differentially Expressed Genes

**Figure 7:**
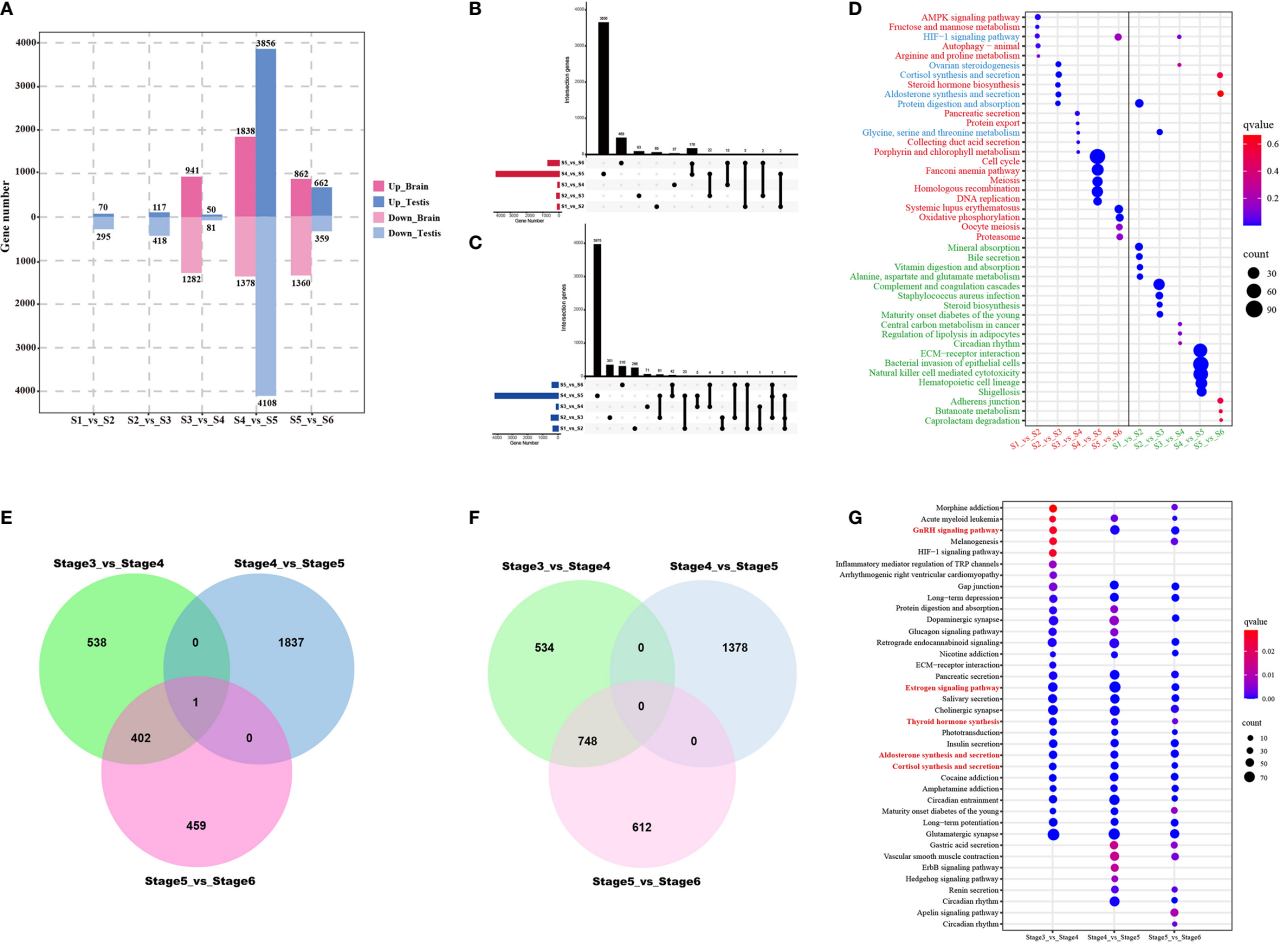
Differentially expressed genes (DEGs) and functional analysis across testicular development from the first stage to the sixth stage. **(A)** Bar chart showing numbers of DEGs between consecutive time points in the brain and the testes. UpSet plots indicate numbers of the common and stage-specific up-regulated **(B)** and down-regulated **(C)** DEGs in the testis. **(D)** KEGG pathway enrichment map showing the top 5 significant pathways of upregulated and downregulated testis-specific DEGs for five stage-by-stage comparisons. Red font, green font, and blue font representing pathways enriched by upregulated DEGs, pathways enriched by downregulated DEGs, pathways enriched by upregulated and downregulated DEGs, respectively. Venn diagram representation of the number of common and stage-specific up-regulated **(E)** and down-regulated **(F)** DEGs in the brain. **(G)** KEGG pathway enrichment map showing the top 5 significant pathways of upregulated and downregulated brain-specific DEGs for three stage by stage comparisons during testis development. Red font, green font, and blue font representing pathways enriched by upregulated DEGs, pathways enriched by downregulated DEGs, pathways enriched by upregulated and downregulated DEGs, respectively.

Identification of stage-specific or -common genes can provide a deeper understanding of the dynamic changes in the developing testes from stage 1 (S1) to stage 6 (S6); therefore, it is also essential to identify differentially expressed genes (DEGs) between adjacent stages (i.e., S1 *vs.* S2, S2 *vs.* S3, S3 *vs.* S4, S4 *vs.* S5, and S5 *vs.* S6) (**Figure 7**). DEGs were to be highly variable among the different development stages. In the testes, the highest number of DEGs were identified between S4 and S5, with 7,964 DEGs, of which 3,856 were up-regulated and 4,108 were down-regulated (**Figure 7A**). The libraries of S1 *vs.* S2, S2 *vs.* S3, S3 *vs.* S4, and S5 *vs.* S6 were found to have 365, 535, 132, and 1,021 DEGs, respectively. In the brain, 7,661 DEGs were identified, including 2,223 between S3 and S4, 3,216 between S4 and S5, 2,222 between S5 and S6 (**Figure 7A**).

In the testes, the number of shared and unique up-regulated and down-regulated DEGs among the five groups is displayed in an UpSet plot (**Figures 7B, C**). Comparisons among all groups revealed that the overlap between the common DEGs among the five groups were zero, irrespective of whether it was an up-regulated or down-regulated group. Only two or three comparison groups had intersected, merely containing a few genes. We further performed KEGG pathway enrichment analysis on stage-specific DEGs for these five comparison groups. The top 5 pathways enriched in up-regulated and down-regulated DEGs were shown in **Figure 7D**. Upregulated DEGs were found to be mainly involved in pathways related to metabolism and steroid hormone synthesis during stage 1 to stage 3, whereas those in cell cycle and meiosis between stage 4 and stage 6. Downregulated DEGs were almost enriched for the metabolism pathways across the time-course of testes development. These stage-specific pathways were in agreement with histology and immunofluorescent analysis, belonging to the characteristics of testes development.

**Figure 8:**
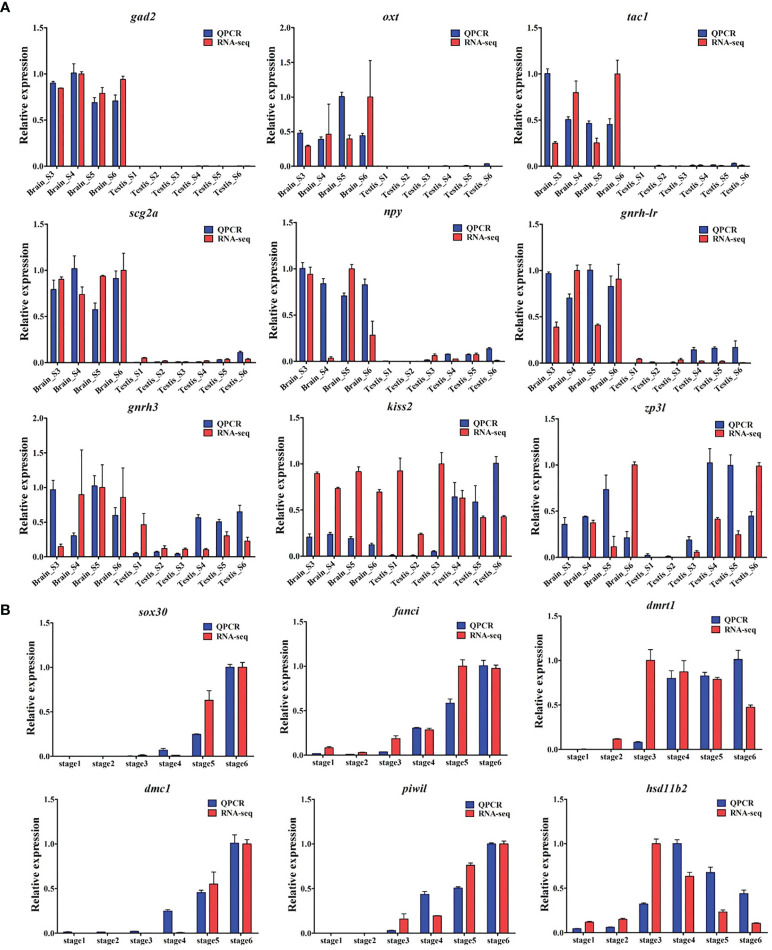
Validation of RNA-seq data using qRT-PCR. In total, expressions of 15 genes were detected by RNA-seq (red column) and qRT-PCR (blue column). **(A)** The expressions of *gad2*, *oxt*, *tac1*, *scg2a*, *npy*, *ghrh-lr*, *gnrh3*, *kiss2*, and *zp3l* genes in the brain and testes at different stages during gonadogenesis in common carp. **(B)** The expressions of *sox30*, *fanci*, *dmrt1*, *dmc1*, *piwil*, and *hsd11b2* genes in the testis across gonad development.

In the brain, the number of common and specific up-regulated and down-regulated DEGs among the three groups were displayed in **Figures 8E, F**. Venn diagram analysis revealed that only one upregulated DEG (*cyp19a1b*) overlapped among the three comparison groups (**Figure 7E**); however, no common downregulated DEG was observed (**Figure 7F**). All DEGs were subjected to enrichment analysis of the KEGG pathway. This analysis resulted in the identification of 32, 41, 39 signaling pathways (*q*-value < 0.05) for S3 *vs.* S4, S4 *vs.* S5, and S5 *vs.* S6, respectively. Among these, five of the top 30 significant pathways of all enriched DEGs were related to endocrine regulation of reproduction, such as the GnRH signaling pathway, estrogen signaling pathway, thyroid hormone synthesis, aldosterone synthesis and secretion, and cortisol synthesis and secretion (**Figure 7G**).

### Validation of Key Genes by qRT-PCR

As shown in **Figure 8**, the expression trends of the 15 selected genes were consistent between the qRT-PCR results and those of the transcriptomic profile analysis, confirming the accuracy and reliability of the RNA-seq. In detail, genes (*gad2*, *oxt*, and *tac1*, etc.) specifically expressed in the brain were compared with those in the testes at different stages of gonad development (**Figure 8A**). Several potential male-biased genes (*fanci* and *sox30*) were corroborated in testis (**Figure 8B**), where the trends of their expressions were in accordance with those of the known genes (*dmrt1*, *dmc1*, and *piwil*) related to testicular differentiation.

## Discussion

*C. carpio* is important in freshwater aquaculture production and is widely studied in genetics, reproduction, and many other fields. A high-quality genome sequence and various genetic tools are available for *C. carpio* in 2014 (16), which was the first reported fish genome. There was room for improvement in the existing genome annotation because it was mainly based on resequencing and data from several bulk RNA-seq (17, 38). However, comprehensive investigations have been restricted by the availability of limited omics data in functional genomics studies relative to the model organism. Therefore, more diverse omics data are required to help enrich and re-annotate the carp genome, especially for vital tissues and their important developmental stages. Availability of precise genome sequence has greatly promoted gene function study and applications in humans (39); however, there is still a long way to go for functional genomic study of *C. carpio*. In the previous study, we have reported the detailed process of early gonadal sex differentiation by combining the expression patterns of sex-related genes (such as *dmrt1*, *sox9b*, and *dmc1*) along with histological changes in *C. carpio* tissues (3). We have also established the timing of gonadal development in *C. carpio* to further explore sex differentiation and gonadal maturation. Here, we generated a time-course transcriptome landscape for the *C. carpio* including the brain and testes throughout the entire development process. Using these data sets, the transcription landscape was systematically explored across 2 tissues and 6 different stages in *C. carpio*. In this study, we have identified 98,345 novel isoforms and 3,071 novel genes compared with those reported in NCBI and Ensembl (**Figures 3A, C**), indicating the extensive usage of isoforms during testes development in the *C. carpio* genome. To date, the functional significance of alternative isoforms and their functions in fish have been explored, with the rare exception of the sox9, *dmrt1*, and *foxl2* (9–11, 40). In total, 25,393 genes with different protein isoforms could result from alternative splicing, alternative promoter usage, alternative initiation, or ribosomal frameshifting (**Figure 3**). This systematic transcriptome study at six important developmental stages in the brain and testes will function as an important resource for *C. carpio* and future reproduction research.

Alternative splicing (AS) is prevalent in both the brain and the testes (41, 42); interestingly, both these are important organs of the HPG axis. The HPG axis is crucial in the reproductive endocrine system in animals. Of the five classic AS events (29), exon skipping (ES) events account for the highest proportion in the brain and testes of *C. carpio* (**Figure 4A**). In order to investigate the functional role of alternative splicing in gonadal development, stage-specific and gonadal-specific alternative splicing patterns between the brain and testes were analyzed (**Figures S3**–**S6**), which revealed hundreds of differentially spliced exons that preferentially alter essential protein domains harboring function-changing mutations. We demonstrated that *robo2*, a member of the *robo* gene family, functions as a regulator of cell migration and tissue morphogenesis in different taxa (43, 44), governing a significant ES event in the brain compared with that in the testes during gonad development (**Figure 4D**). A recent study has reported that *robo2* regulated synaptic oxytocin content by affecting actin dynamics (45), demonstrating that this gene performed has an *in situ* testicular function using testis-specific exons in *C. carpio*. Take another example, the *epb41l3b* gene maintains testicular maturation by suppressing two exons at stage 6 of development in the testes compared to that in the brain (**Figure 4E**). The *epb41l3b* gene encoding erythrocyte membrane protein band 4.1 like 3b, also known as protein 4.1B, is necessary for glutamatergic synapse formation in the sensorimotor circuit of developing zebrafish (46). A similar scenario has also been observed found in *D. rerio* (PRJNA557435) between expression in the brain and gonad (**Figure 4F**). Our results implied that the ES event of the *epb41l3b* gene is evolutionarily conserved in fishes. Taken together, we described the alternative splicing landscape and dynamic changes in both the brain and testes during gonad development. These results provide an important foundation to further decode the molecular mechanism of these tissue-specific and stage-specific isoforms during reproduction.

Although the role of HPG axis in inducing testes development has been studied well in fish, the key signal pathway co-expressed by the gonad and brain remains to be elucidated. In this study, 23 samples from the brain and gonad were collected at six different development stages to generate transcript data (**Figure 6**). Vital signaling that induces testes development was identified by performing a WGCNA, which incorporates high-dimensional transcriptome data into fewer variables by constructing different co-expression modules and provides further insights into the relationships between modules and stage phenotypes (47, 48). The “lightcyan” module was identified as the critical module that is significantly related to the brain and gonad samples (**Figure 6A**). Subsequently, the function and pathway enrichment analysis of the critical module closely related to testes development was performed (**Figures 6C, D**). The crossover of GO terms and KEGG pathways results in ubiquitin-mediated proteolysis (**Figures 6C, D**), which is essentially required throughout all developmental stages of mammalian spermatogenesis (49, 50). This has also been reported in flatfish (51). Our results implied that it might also be necessary for testes development in *C. carpio*. Notably, a critical gene (zona pellucida sperm-binding protein 3-like, *zp3l*) was identified as the hub gene of the ‘lightcyan’ module (**Figure 6E**). Previous studies have reported that *zp3l* is a male-biased gene, located in the zona pellucida, and is essential in sperm binding and zona matrix formation (52–54). Moreover, *zp3l* has been identified as a reproduction-related gene (55), which exhibits inhibition by gonadotropin stimulation (56). These results broaden existing understanding of signaling in the brain and testes that regulates gonad development, thereby providing a new research perspective for reproductive regulation research.

**Figure 9:**
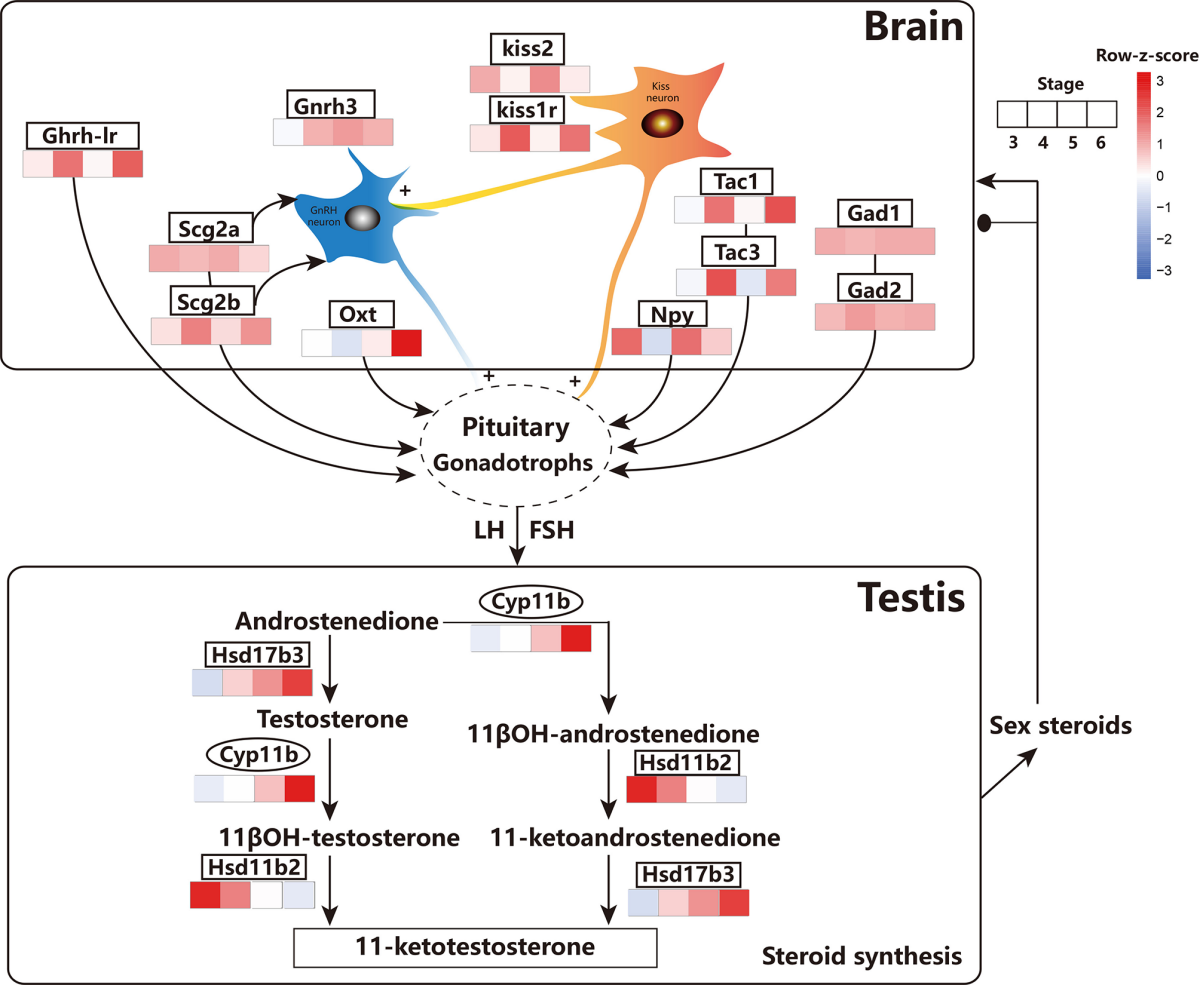
Schematic diagram showing the main events related to the testis and brain involved in the HPG axis across testicular differentiation and maturation inferring from the results of this study. GnRH3, Gonadotropin-releasing hormone; Kiss2, Kisspeptin2; Kiss1r, Kisspeptin1r; GHRH-LR, pituitary adenylate cyclase-activating polypeptide type I receptor-like; SCG2a/b, Secretoneurin II a/b; OXT, isotocin; GAD1/2, γ-aminobutyric acid; NPY, neuropeptide Y; TAC1/3, Tachykinin 1/3; Cyp11b, Cytochrome P450 family 11 subfamily B polypeptide; Hsd17b2, Hydroxysteroid 17-Beta dehydrogenase B2; Hsd17b3, Hydroxysteroid 17-Beta dehydrogenase B3. FSH, follicle-stimulating hormone; LH, luteinizing hormone. the transcription factor. The expression values were represented by FPKM/z-score. Ovals represent the transcription factors. The GnRH in the brain stimulates the secretion of pituitary gonadotropins, FSH, and LH, which in-turn act on the testes to stimulate the production of sex steroid hormones.

Fish reproduction is usually regulated by the precise coordination of neuroendocrine hormones, acting on the HPG axis through a cascade of key genes (57). Typically, three distinct isoforms of GnRH (GnRH1, GnRH2, and GnRH3) co-exist in modern teleosts (58). In this study, *gnrh2* and *gnrh3* were identified in the brain, which were highly expressed during spermatogenesis (stage 4 and stage 5) and spermiation (stage 6) compared with that in the pre-differentiation stage (stage 3) of the testes, which is consistent with previous reports (59–61). Furthermore, both kisspeptin (*kiss2*) and its receptor (*kiss1r*) were also found to be dynamically altered during testicular differentiation and maturation (**Figure 9**). Kisspeptins are a family of structurally related peptides encoded by the *kiss* gene that have the ability to activate the G-protein coupled receptor 54. They have been shown to play be crucial in regulating the gonadotropic axis and function as the ‘‘gatekeeper” of puberty in fish (8, 62, 63). *Kiss2* levels were found to gradually increase from the immature stage (stage 4) to spermiation (stage 6), reaching its maximum level during late spermatogenesis (stage 5) (**Figure 9**). Whole-cell patch-clamp analysis revealed that kisspeptin (*kiss2*) can directly affect *gnrh3* neurons in the brain (64, 65). Additionally, kiss neurons could receive and then transmit steroid feedback to the GnRH neurons (66, 67). Critical factors in the Kiss-KissR-GnRH systems were identified, and dynamic changes were described during testicular development, providing new time-course omics data for endocrinology studies.

In addition to classic neurotransmitters, recent investigations have focused on the roles of other substances in teleost reproduction. For instance, glutamate, γ-aminobutyric acid, and serotonin have been firmly verified to have significant effects on hypothalamic-pituitary function in teleosts (8). In order to explore new candidates, dynamic changes in *oxt*, *scg2a*, *scg2b*, *gad1*, *gad2*, *tac1*, *tac3*, *npy*, and *ghrh-lr* in the brain and testes of common carp were monitored during testicular development. Interestingly, these genes were all specifically expressed in the brain; however, they were hardly expressed in the testes at different developmental stages (**Figure 9**). In detail, the expression of *oxt* significantly increased during the full spermatogenesis (stage 4 and stage 5) and spermiation (stage 6). Isotocin (*oxt*) is the teleost homologue of mammalian oxytocin and a reproductive neuropeptide that is regulated by multiple neurotransmitter systems (68, 69). The expression of the secretory granule protein secretogranin-2 (*scg2a* and *scg2b*), processed into the bioactive neuropeptide secretoneurin that affects the release of pituitary luteinizing hormone (70), was higher in the brain at all stages of testicular differentiation. The expressions of *gad1* and *gad2*, amino acid neurotransmitters that regulate *lh* release (71), were consistently high from the beginning of testicular differentiation (stage 4) until the onset of puberty (stage 6). The expressions of the *tac1* and *tac3* genes were higher at the beginning of spermatogenesis (stage 4) and the onset of puberty (stage 6) compared with those in the undifferentiation period (stage 3) and at the end of spermatogenesis (stage 5). Tachykinin are a family of neuropeptides, including neurokinin A (NKA) and neurokinin B (NKB), which are encoded by the *tac1* gene and the *tac2/3* gene, respectively. Recent studies have shown that NKB and kisspeptin are co-expressed in the brain and function as critical signals in the suite of neurochemical inputs that activate the reproductive axis at puberty (72). This indicates the potential role of *tac1* and *tac3* genes in neuroendocrine control of puberty and reproduction in common carp. The expression of *npy* was at its the lowest level at the beginning of spermatogenesis compared with that at other stages. In goldfish, *npy* has been shown to be directly involved in the release of *lh* at the level of gonadotrophs in the pituitary or indirect release of GnRH from GnRH terminals in the pituitary or preoptic region slices (8). A similar regulation mechanism might occur in common carp during late spermatogenesis and spermiation. The expression of *ghrh-lr* significantly increased at the beginning of spermatogenesis (stage 4) and the onset of puberty (stage 6). The *ghrh-lr* gene, encoding the pituitary adenylate cyclase-activating polypeptide type I receptor-like, can regulate the release of *lh* and be involved in spermatogenesis and sperm motility (73). Therefore, multiple candidates in the brain of the HPG axis were identified to be differentially regulated by various neurotransmitter systems from the progression of testicular development (**Figure 9**).

In the testes, these neurotransmitters can trigger a series of syntheses and secretion of sex steroids regulated by the HPG axis. Moreover, sex steroids produced by the testes feedback both positively and negatively to the brain, which in-turn finely controls gonadotropin secretion (74). In this study, several genes involved in steroidogenesis in the testes of common carp were identified, including cytochrome P450 family 11 subfamily b (*cyp11b*), hydroxysteroid 11β-dehydrogenase 2 (*hsd11b2*), and hydroxysteroid 17-β dehydrogenase 3 (*hsd17b3*) (**Figure 9**). Among these, *cyp11b* converts androstenedione into 11β-OH-androstenedione and testosterone to 11βOH-testosterone (75, 76). The transcript levels of the *cyp11b* gene increased in early spermatogenesis (stage 4) and peaked the beginning of puberty (stage 6), which is in agreement with a previous report on male gonad development in Silver sillago (77) and rainbow trout (78). This demonstrated that *cyp11b* is critical in promoting testicular development in common carp.

Collectively, time-course transcriptomics was combined with histology and immunofluorescent analysis to describe a comprehensive landscape of alternative splicing events and gene expression during gonadogenesis in common carp. Furthermore, several potential male-preference genes (*fanci* and *sox30*) and brain-specific genes (*oxt*, *gad2*, and *tac1*, etc.) related to the HPG axis were identified in the testes and brain of common carp, respectively. These findings provide insights that are essential to decipher the mechanisms underlying testis development in Cyprinidae as well as other teleosts. However, the functions and biogenesis of stage-specific and tissue-specific isoforms/genes during testes development are both aspects that warrant further investigation.

## Data Availability Statement

The data presented in the study are deposited in the NCBI SRA repository, accession number PRJNA781298.

## Ethics Statement

The animal study was reviewed and approved by Management and Use of Laboratory Animals of Hubei Province. Written informed consent was obtained from the owners for the participation of their animals in this study.

## Author Contributions

DL and WH conceived the project. DL, YZ, and JC designed the study. YZ and KC performed most of the experiment and analyses. FL, MJ, and HC helped in experiment, data curation, and analyses. YL helped in breeding and sampling. ZC, YS, BT, and XC helped in analyzing the image data. YZ, DL, WH, JC, and ZZ prepared the draft and final version of the manuscript. All authors contributed to the article and approved the submitted version.

## Funding

This work was supported by grants from the Strategic Priority Research Program of CAS (Grant No. XDA24010108) to WH and DL, the National Natural Science Foundation of China (Grant No.31922085 to DL, Grant No.31721005, 32030113 to WH), and Natural Science Foundation of Hubei Province (Grant No. 2020CFA056 to DL). China Postdoctoral Science Foundation on the 70th finance (Grant No. 2021M703436 to YZ).

## Conflict of Interest

The authors declare that the research was conducted in the absence of any commercial or financial relationships that could be construed as a potential conflict of interest.

## Publisher’s Note

All claims expressed in this article are solely those of the authors and do not necessarily represent those of their affiliated organizations, or those of the publisher, the editors and the reviewers. Any product that may be evaluated in this article, or claim that may be made by its manufacturer, is not guaranteed or endorsed by the publisher.

## Supplementary Material

Supplementary Figure S1 Crossing scheme to obtain all-male Yellow River carp. XY all-male carp were produced by crossing the XX normal females with YY super-male carp which were produced using artificial androgenesis (Jiang et al., 2018). Blue, red and purple outer circles denote XY male, XX female, YY super-male common carp, respectively.

Supplementary Figure S2 *de novo* assembly of common carp genome through the transcriptome of the testes and the brain at six different developmental stages. **(A)** Distribution of the number of isoforms per gene. **(B)** The number of transcripts of different splicing types compared with the NCBI annotation. **(C)** Heatmap showing expression profiles of annotated transcripts.

Supplementary Figure S3 The case of the SE pattern of *cadm3* gene and validation using RT-PCR in testis and brain of *C. carpio*. The expression of the *cadm3* exons is shown with sequence coverage depth on the gene loci.

Supplementary Figure S4 The case of the SE pattern of *cadm4* gene and validation using RT-PCR in testis and brain of *C. carpio*. The expression of the *cadm4* exons is shown with sequence coverage depth on the gene loci.

Supplementary Figure S5 The case of the SE pattern of *macf1* gene and validation using RT-PCR in testis and brain of *C. carpio*. The expression of the *macf1* exons is shown with sequence coverage depth on the gene loci.

Supplementary Figure S6 The case of the SE pattern of *ptprfa* gene and validation using RT-PCR in testis and brain of *C. carpio*. The expression of the *ptprfa* exons is shown with sequence coverage depth on the gene loci.

Supplementary Figure S7 The case of the SE pattern of *cast* gene and validation using RT-PCR in testis and brain of *C. carpio*. The expression of the cast exons is shown with sequence coverage depth on the gene loci.

Supplementary Figure S8 The case of the SE pattern of *hipk1a* gene and validation using RT-PCR in testis and brain of *C. carpio*. The expression of the hipk1a exons is shown with sequence coverage depth on the gene loci.

Supplementary Figure S9 The case of the SE pattern of *dmxl2* gene and validation using RT-PCR in testis and brain of *C. carpio*. The expression of the dmxl2 exons is shown with sequence coverage depth on the gene loci.

Supplementary Figure S10 The case of the SE pattern of clasp2 gene and validation using RT-PCR in testis and brain of *C. carpio*. The expression of the *clasp2* exons is shown with sequence coverage depth on the gene loci.

Supplementary Figure S11 The case of the SE pattern of *nrf2b* gene and validation using RT-PCR in testis and brain of *C. carpio*. The expression of the nrf2b exons is shown with sequence coverage depth on the gene loci.

Supplementary Figure S12 The FPKM expression profiles of all genes during testis development.

Supplementary Figure S13 The cumulative fraction curves of the gene expression of 26 samples.

Supplementary Figure S14 Clustering of expression profiles in the brain. **(A)** Heatmap of five expression clusters across gonad development. The bar plots on the right show selected GO enrichment of relevant clusters. BP: biological process; CC: cell component; MF: molecular function. **(B)** Statistic of gene numbers of five clusters in brain; **(C–G)** Time-series expression profile of five clusters. The blue represents the average expression of the cluster, and the background lines represent all genes assigned to this cluster. The expression values were represented by FPKM/z-score.

Supplementary Figure S15 Cluster dendrogram of 23 samples following elimination of Testis_St1_3, Brain_St5_3, and Brain_St6_1.

Supplementary Figure S16 The screen of soft thresholding before weighted gene co-expression network analysis.

Supplementary Figure S17 The analysis of eigengene dendrogram of the module-trait relationship with 23 samples.

Supplementary Figure S18 The heatmap showing the module-trait relationship with 23 samples.
