# Supplementary material for: Dynamic Gene Expression and Alternative Splicing Events Demonstrate Co-Regulation of Testicular Differentiation and Maturation by the Brain and Gonad in Common Carp
Source: Front Endocrinol (Lausanne). 2022 Feb 8;12:820463. doi: 10.3389/fendo.2021.820463 (PMC8867607; doi:10.3389/fendo.2021.820463)
Supplement: Supplementary Figure S1 — Crossing scheme to obtain all-male Yellow River carp. XY all-male carp were produced by crossing the XX normal females with YY super-male carp which were produced using artificial androgenesis (Jiang et al., 2018). Blue, red and purple outer circles denote XY male, XX female, YY super-male common carp, respectively. [file DataSheet_1.docx]

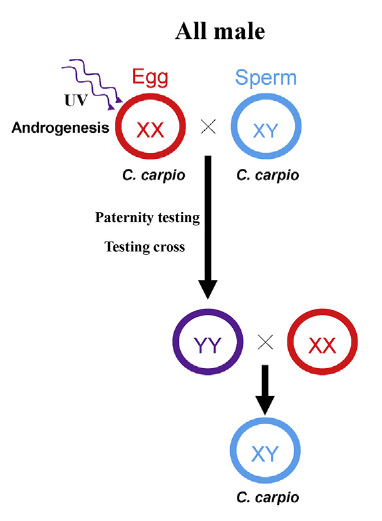


**Fig. S1** Crossing scheme to obtain all-male Yellow River carp. XY all-male carp were produced by crossing the XX normal females with YY super-male carp which were produced using artificial androgenesis (Jiang et al., 2018). Blue, red and purple outer circles denote XY male, XX female, YY super-male common carp, respectively.


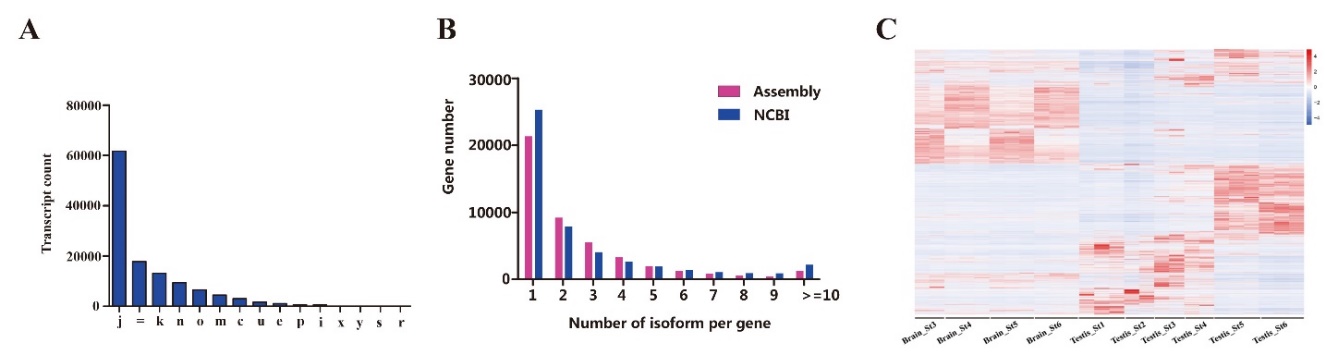


**Fig. S2** *de novo* assembly of common carp genome through the transcriptome of the testes and the brain at six different developmental stages. (A) Distribution of the number of isoforms per gene. (B) The number of transcripts of different splicing types compared with the NCBI annotation. (C) Heatmap showing expression profiles of annotated transcripts.


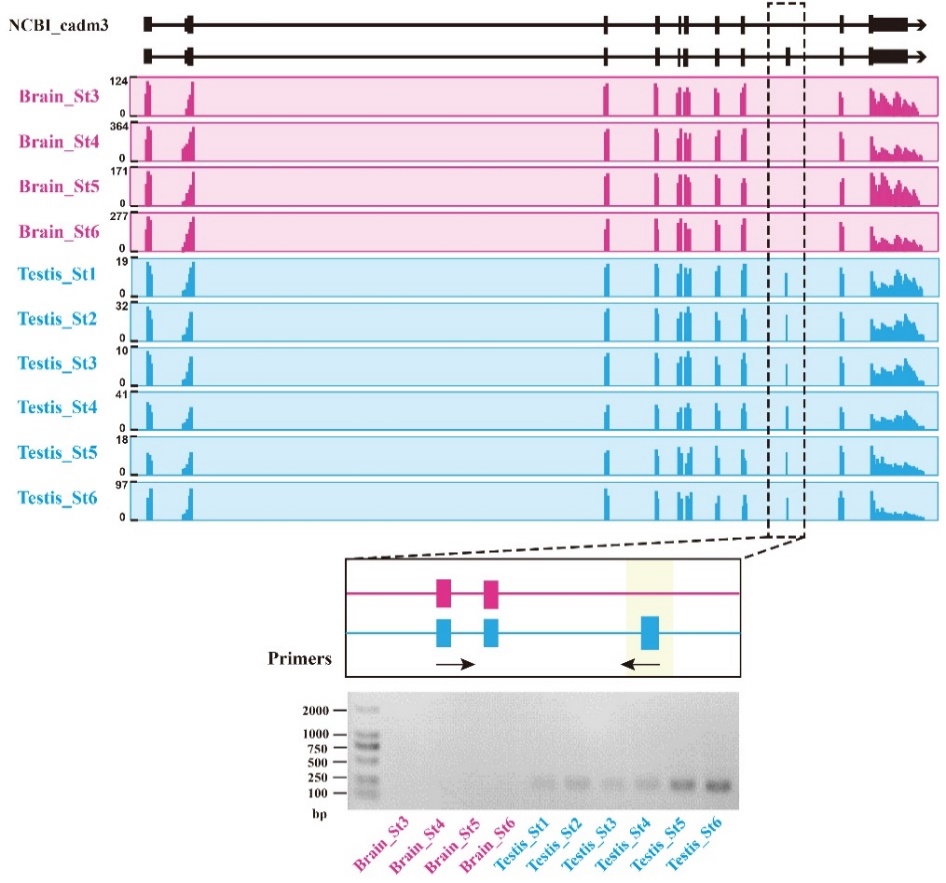


**Fig. S3** The case of the SE pattern of *cadm3* gene and validation using RT-PCR in testis and brain of *C. carpio*. The expression of the *cadm3* exons is shown with sequence coverage depth on the gene loci.


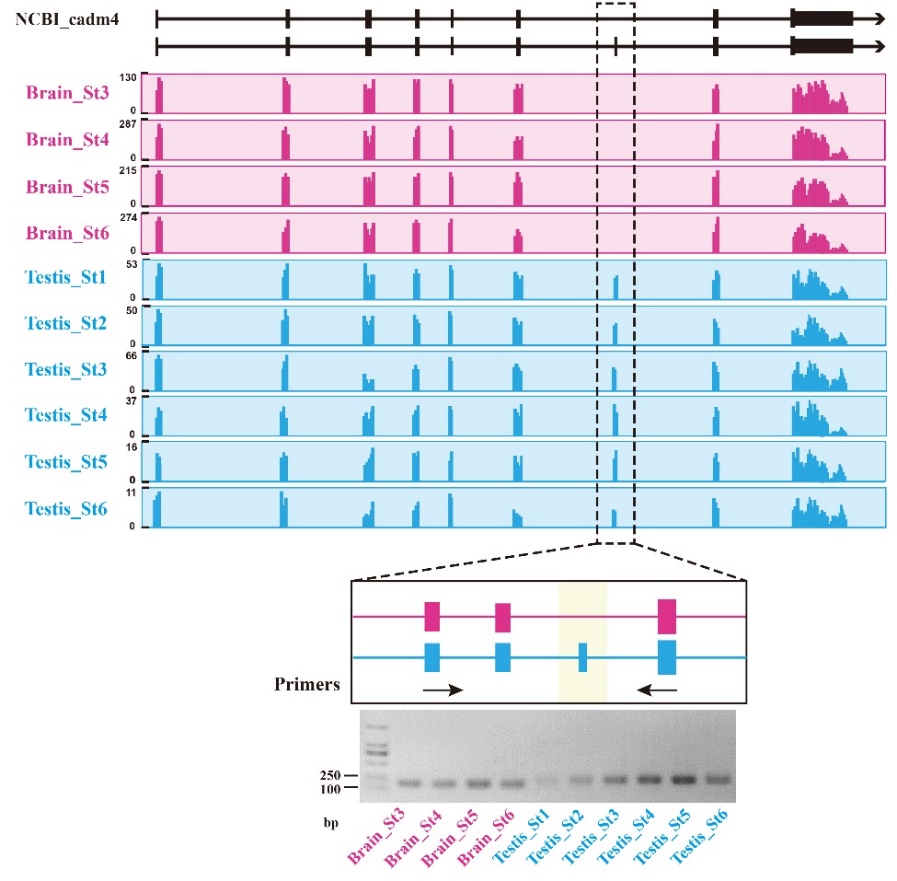


**Fig. S4** The case of the SE pattern of *cadm4* gene and validation using RT-PCR in testis and brain of *C. carpio*. The expression of the *cadm4* exons is shown with sequence coverage depth on the gene loci.


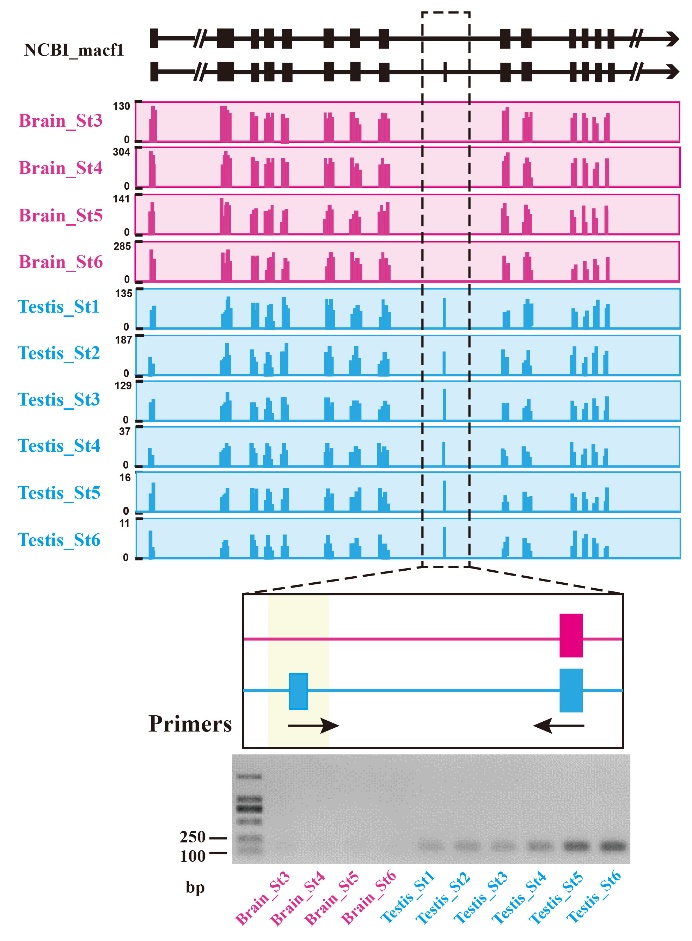


**Fig. S5** The case of the SE pattern of *macf1* gene and validation using RT-PCR in testis and brain of *C. carpio*. The expression of the *macf1* exons is shown with sequence coverage depth on the gene loci.


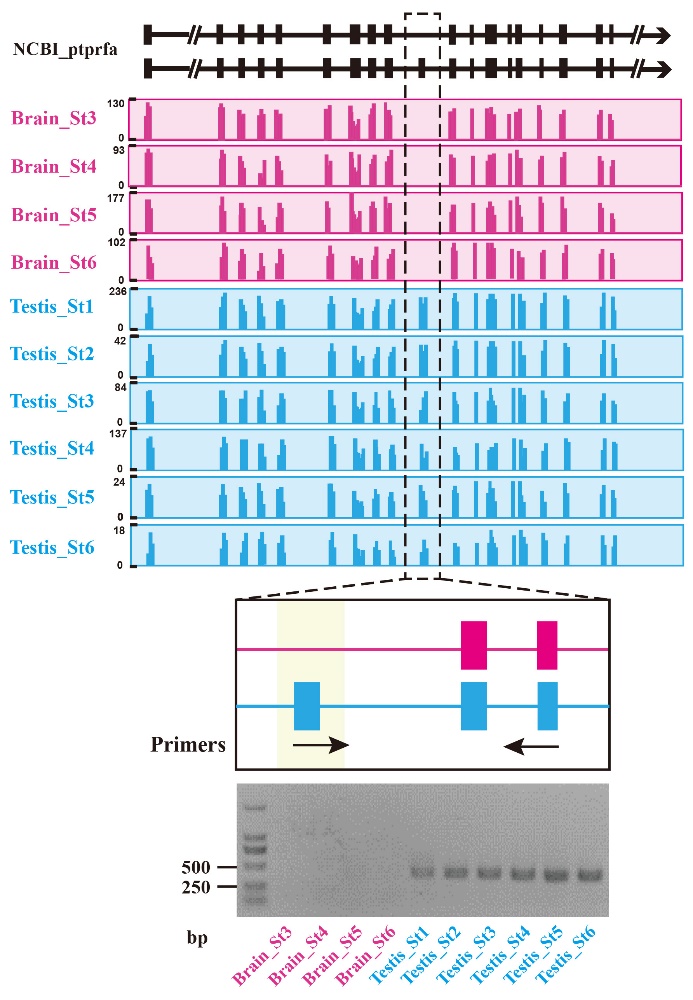


**Fig. S6** The case of the SE pattern of *ptprfa* gene and validation using RT-PCR in testis and brain of *C. carpio*. The expression of the *ptprfa* exons is shown with sequence coverage depth on the gene loci.


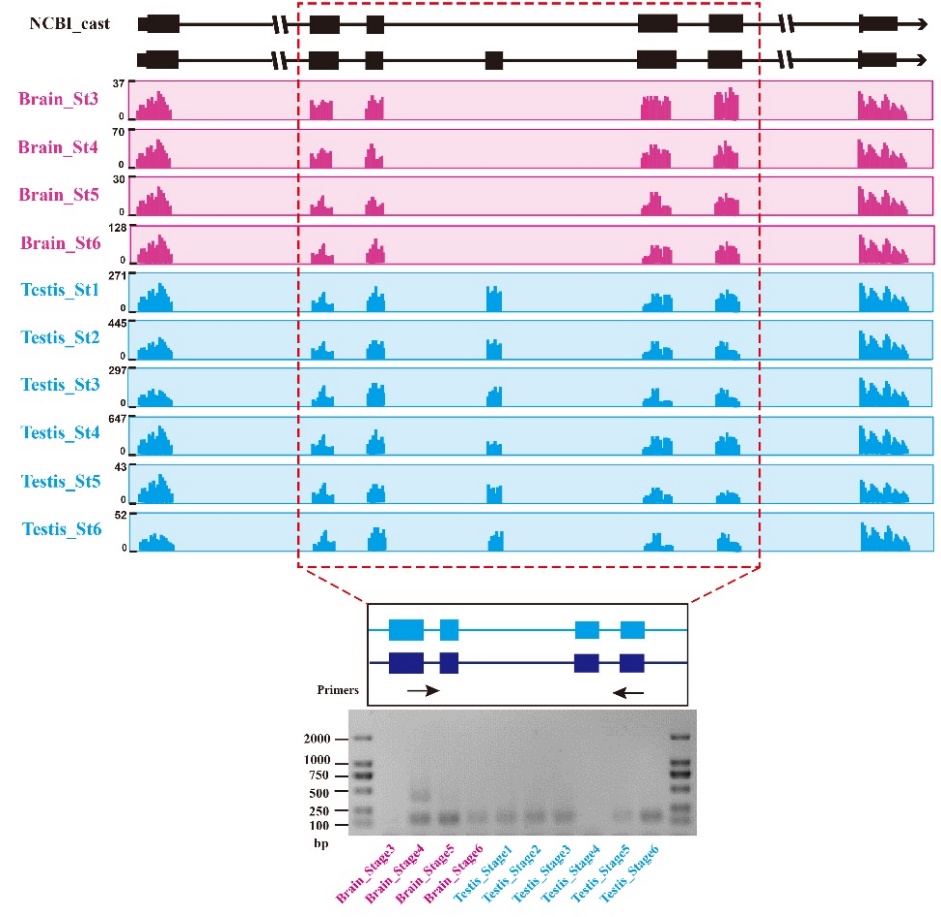


**Fig. S7** The case of the SE pattern of *cast* gene and validation using RT-PCR in testis and brain of *C. carpio*. The expression of the *cast* exons is shown with sequence coverage depth on the gene loci.


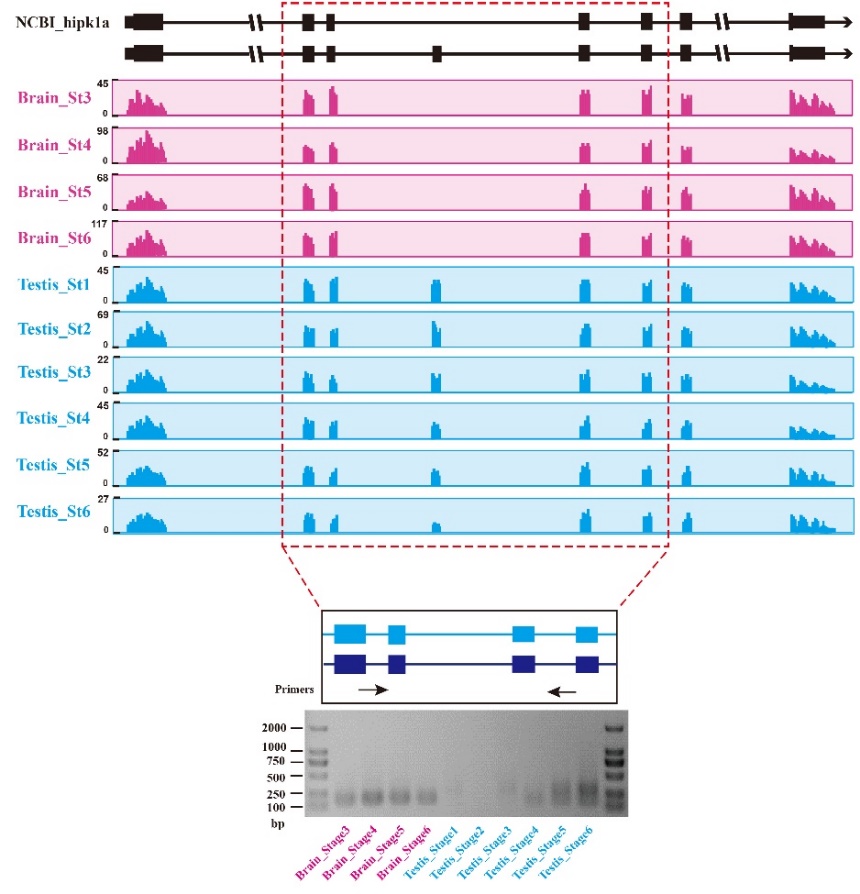


**Fig. S8** The case of the SE pattern of *hipk1a* gene and validation using RT-PCR in testis and brain of *C. carpio*. The expression of the *hipk1a* exons is shown with sequence coverage depth on the gene loci.


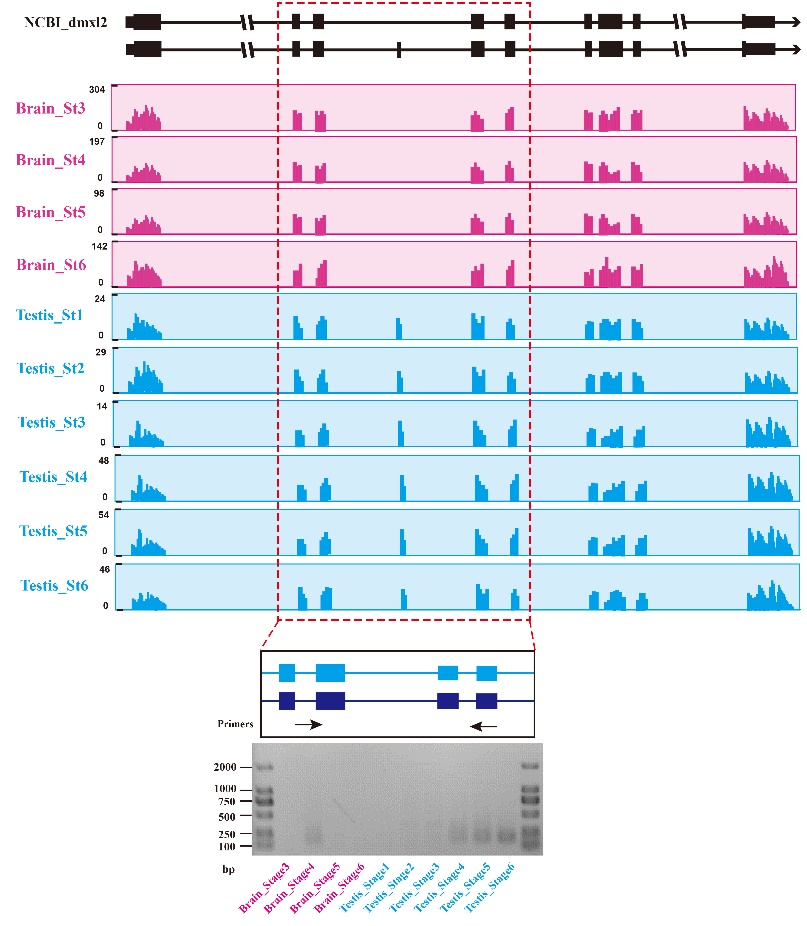


**Fig. S9** The case of the SE pattern of *dmxl2* gene and validation using RT-PCR in testis and brain of *C. carpio*. The expression of the *dmxl2* exons is shown with sequence coverage depth on the gene loci.


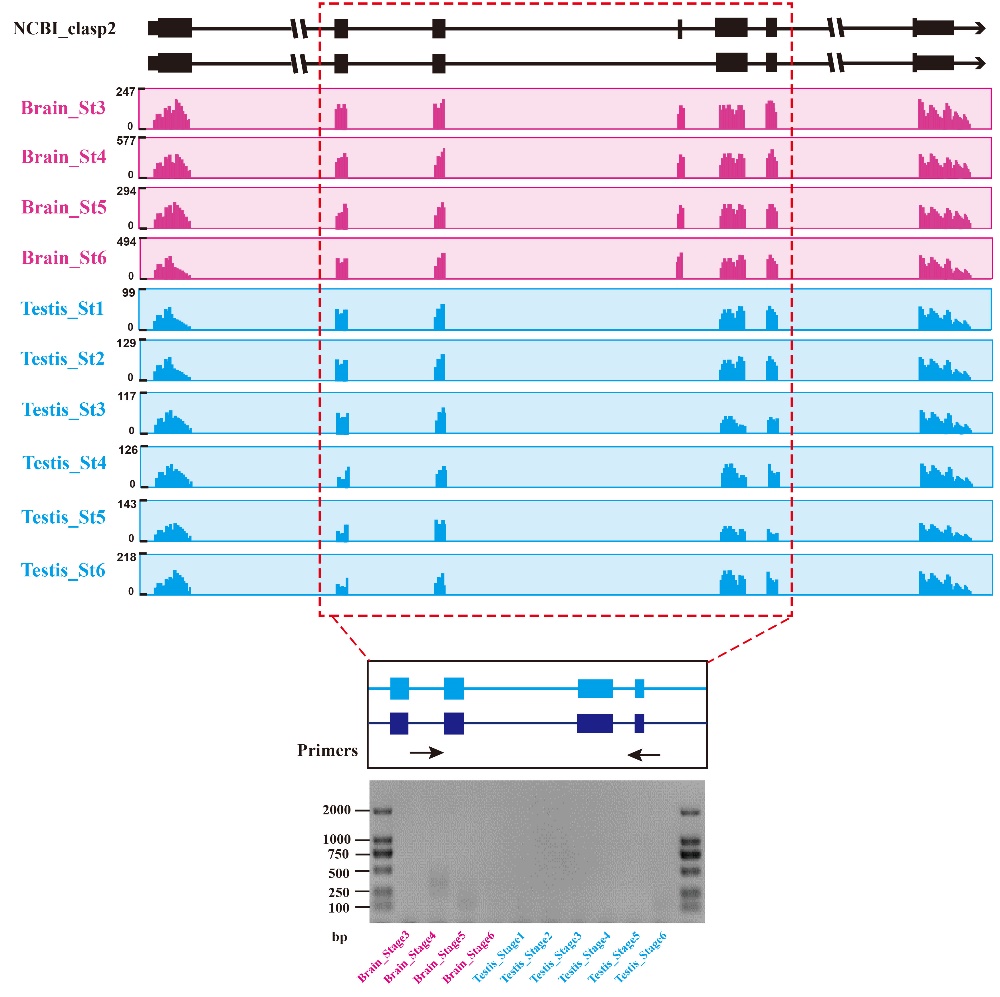


**Fig. S10** The case of the SE pattern of *clasp2* gene and validation using RT-PCR in testis and brain of *C. carpio*. The expression of the *clasp2* exons is shown with sequence coverage depth on the gene loci.


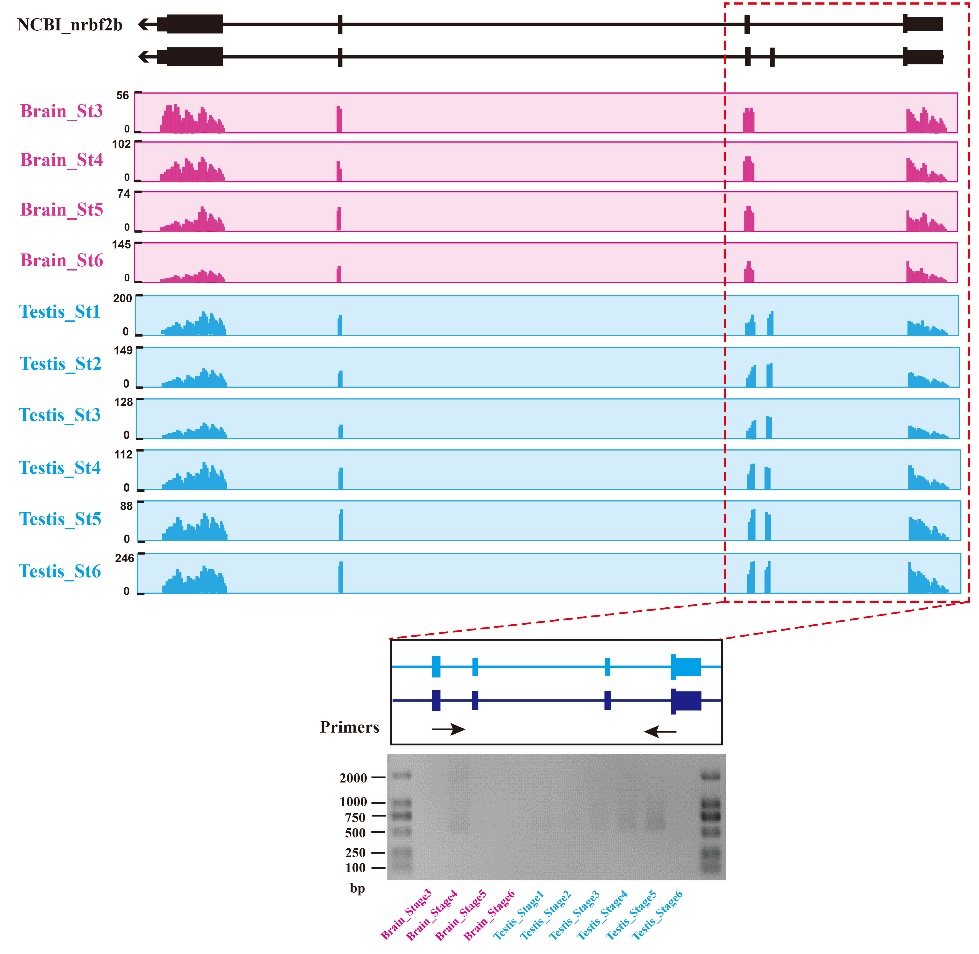


**Fig. S11** The case of the SE pattern of *nrf2b* gene and validation using RT-PCR in testis and brain of *C. carpio*. The expression of the *nrf2b* exons is shown with sequence coverage depth on the gene loci.


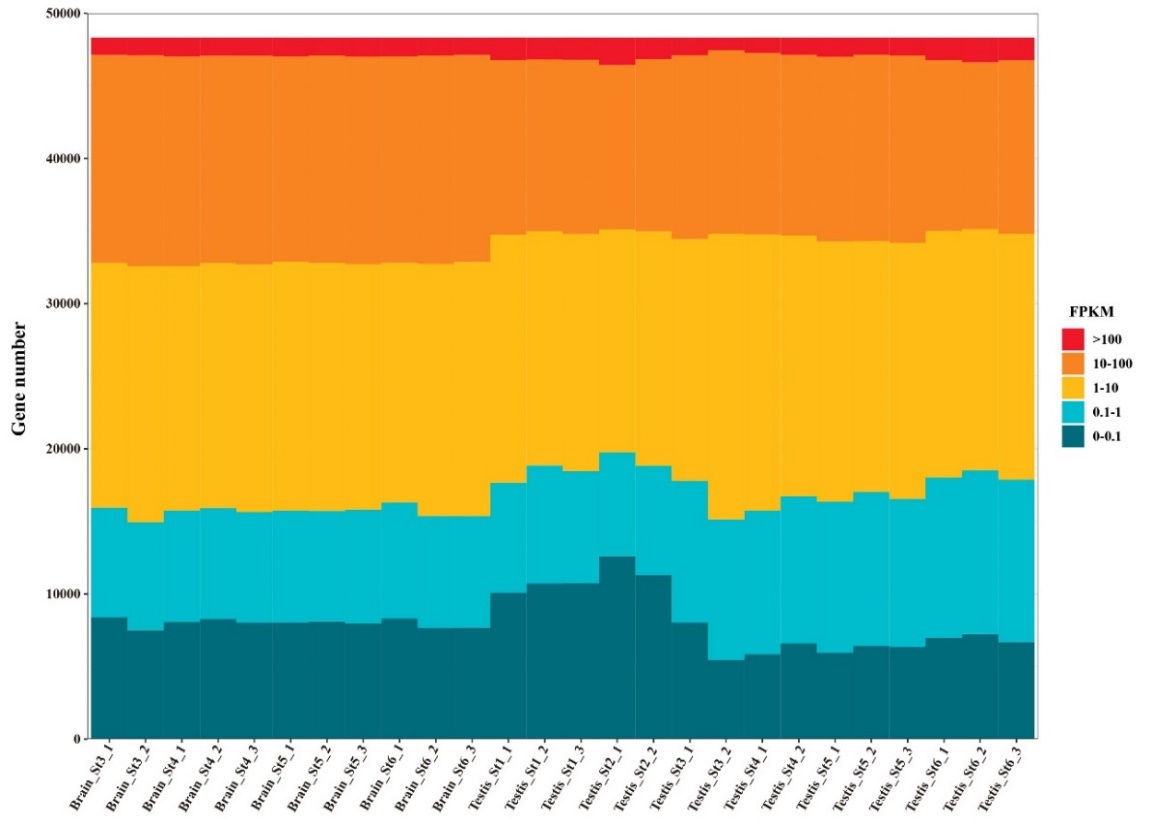


**Fig. S12** The FPKM expression profiles of all genes during testis development.


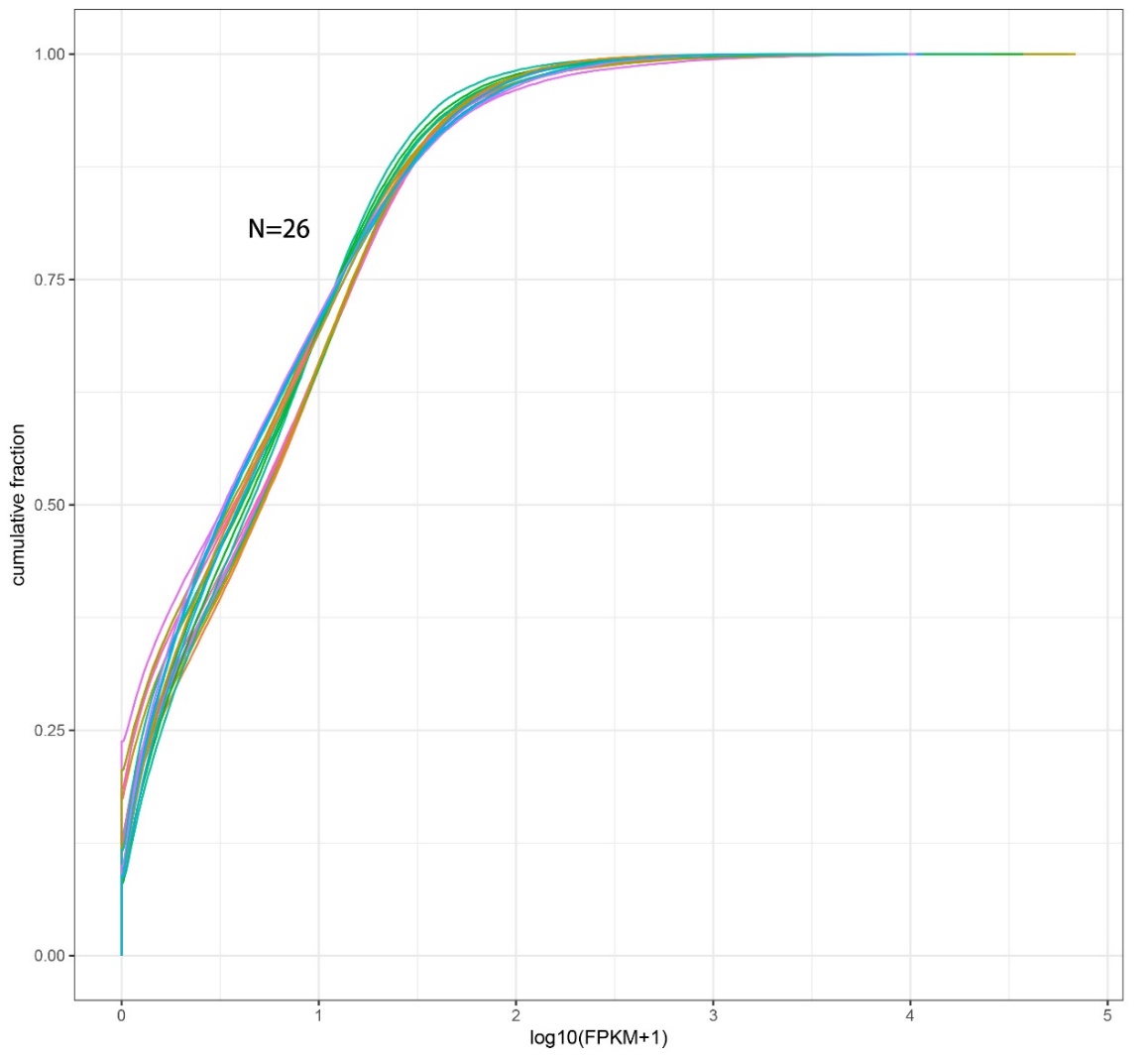


**Fig. S13** The cumulative fraction curves of the gene expression of 26 samples.


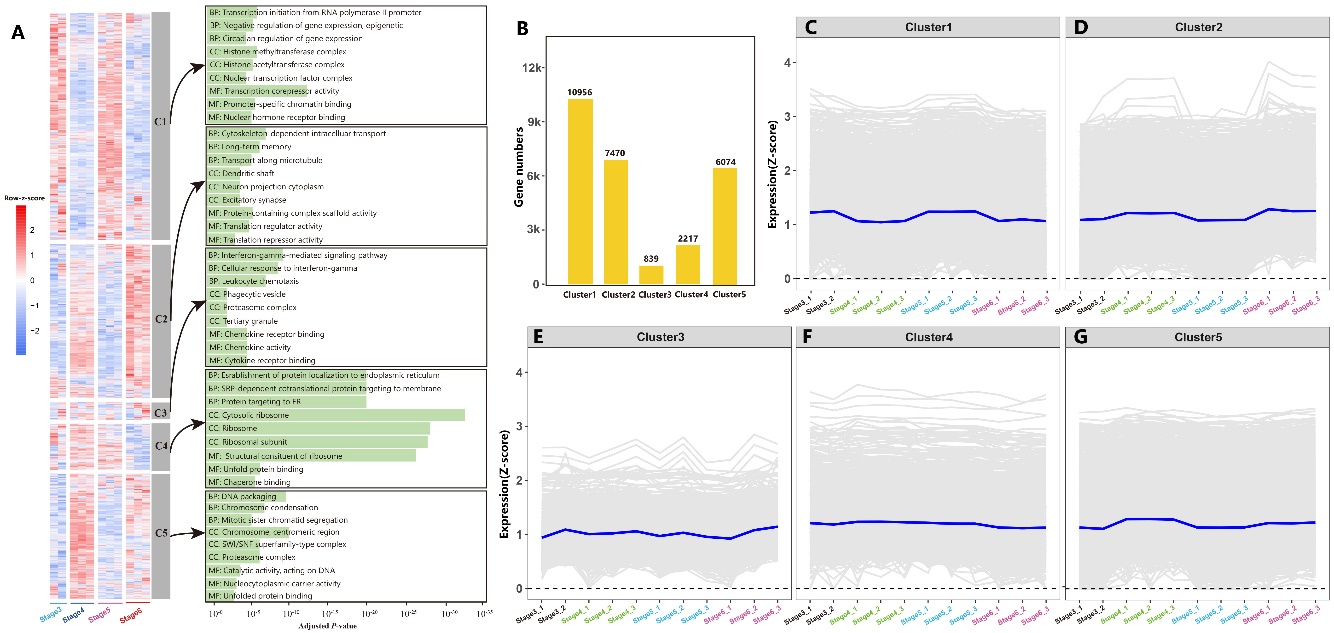


**Fig. S14** Clustering of expression profiles in the brain. (A) Heatmap of five expression clusters across gonad development. The bar plots on the right show selected GO enrichment of relevant clusters. BP: biological process; CC: cell component; MF: molecular function. (B) Statistic of gene numbers of five clusters in brain; (C-G) Time-series expression profile of five clusters. The blue represents the average expression of the cluster, and the background lines represent all genes assigned to this cluster. The expression values were represented by FPKM/z-score.


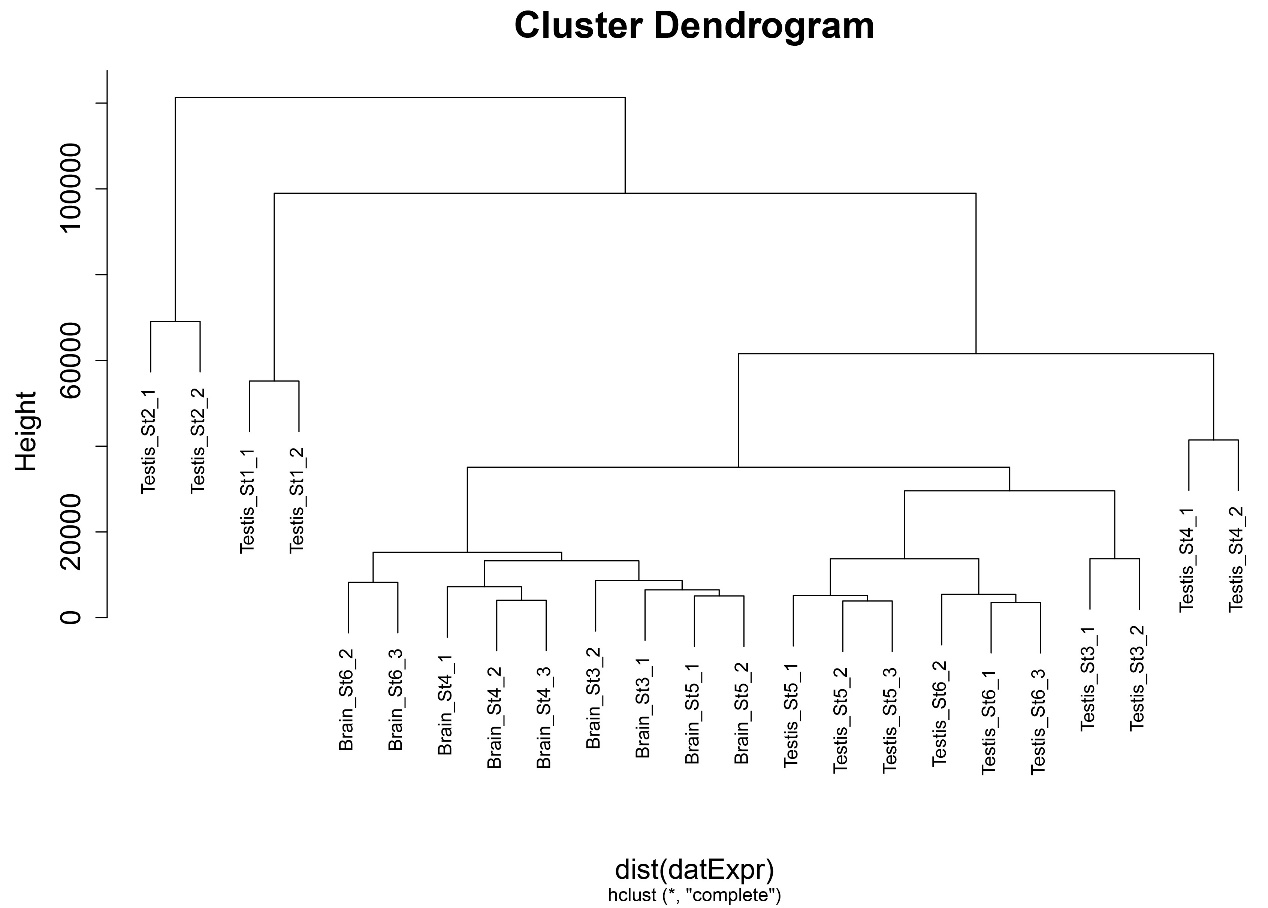


**Fig. S15** Cluster dendrogram of 23 samples following elimination of Testis_St1_3, Brain_St5_3, and Brain_St6_1.


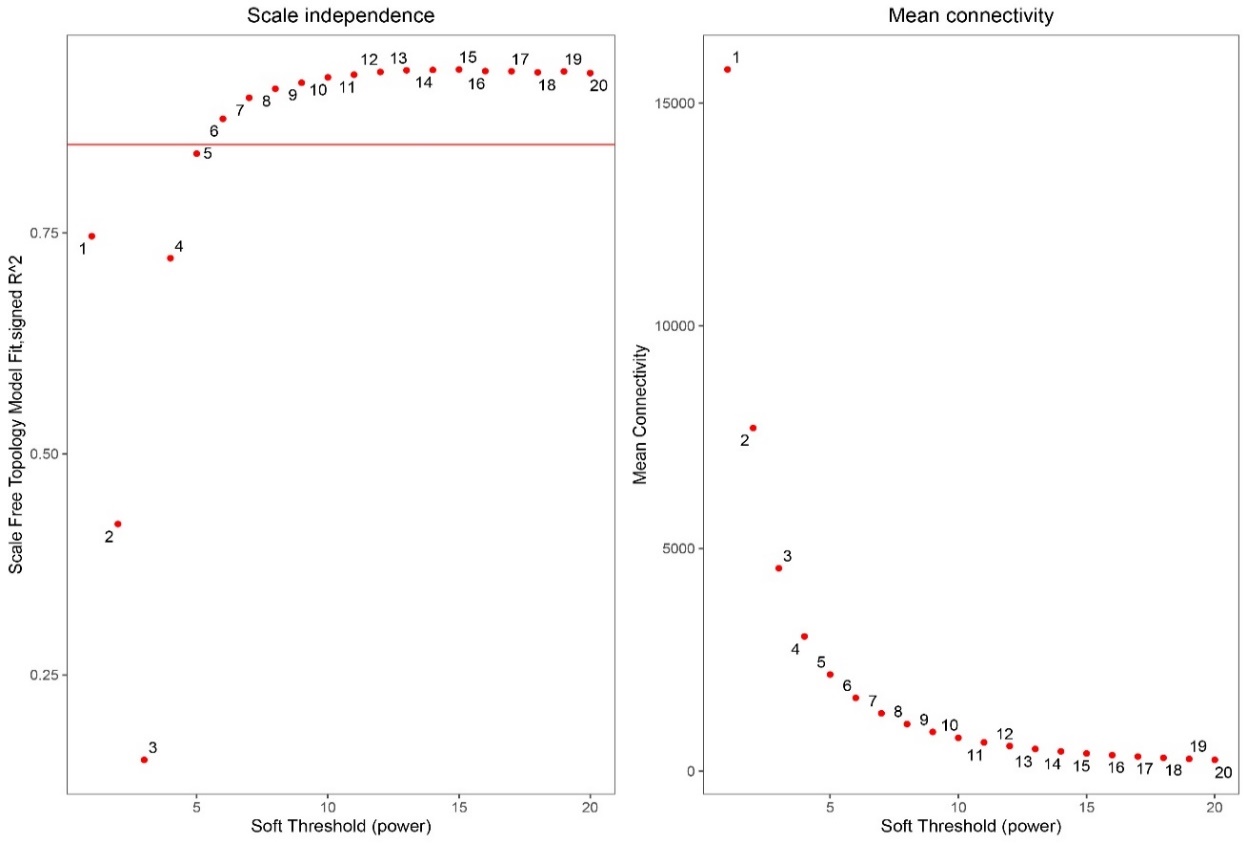


**Fig. S16** The screen of soft thresholding before weighted gene co-expression network analysis.


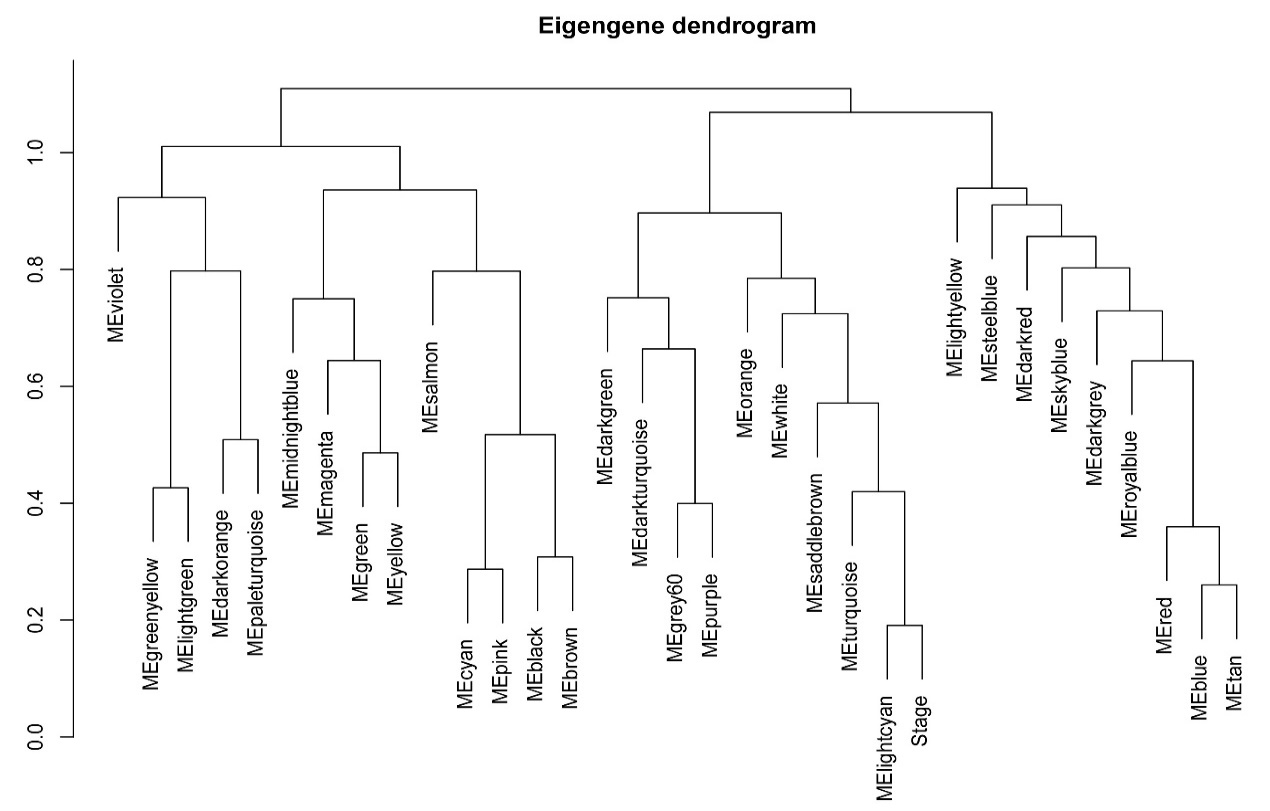


**Fig. S17** The analysis of eigengene dendrogram of the module-trait relationship with 23 samples.


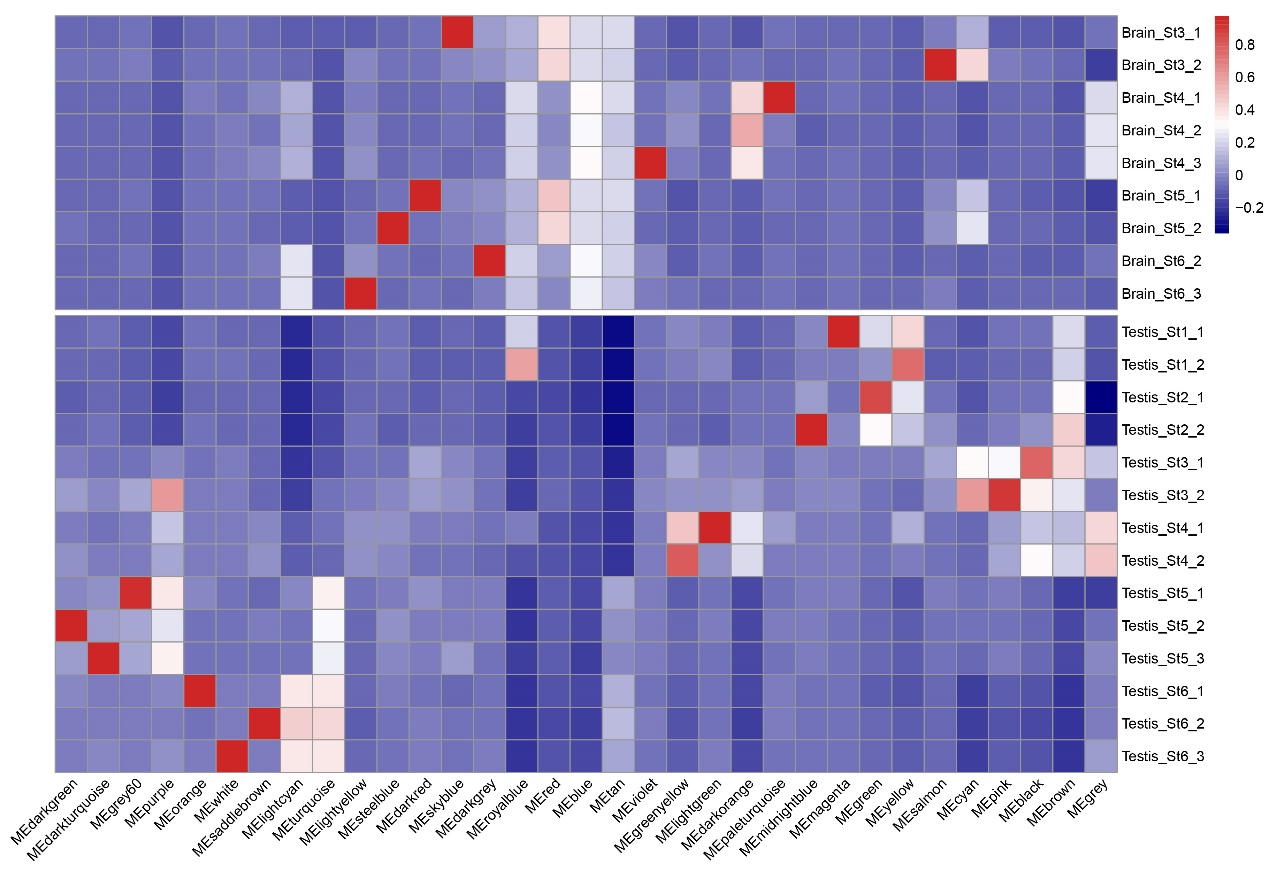


**Fig. S18** The heatmap showing the module-trait relationship with 23 samples.
